# Dynamic interaction of poly(A)-binding protein with the ribosome

**DOI:** 10.1038/s41598-018-35753-1

**Published:** 2018-11-28

**Authors:** Kodai Machida, Tomoaki Shigeta, Yuki Yamamoto, Takuhiro Ito, Yuri Svitkin, Nahum Sonenberg, Hiroaki Imataka

**Affiliations:** 1 Department of Applied Chemistry, Graduate School of Engineering, University of Hyogo, Himeji, 671-2201 Japan; 2 Division of Structural and Synthetic Biology, RIKEN Center for Life Science Technologies, and Laboratory for Translation Structural Biology, RIKEN Center for Biosystems Dynamics Research, Yokohama, 230-0045 Japan; 3 Department of Biochemistry and Rosalind and Morris Goodman Cancer Research Centre, McGill University, Montreal, H3A 1A3 Canada

## Abstract

Eukaryotic mRNA has a cap structure and a poly(A) tail at the 5′ and 3′ ends, respectively. The cap structure is recognized by eIF (eukaryotic translation initiation factor) 4 F, while the poly(A) tail is bound by poly(A)-binding protein (PABP). PABP has four RNA recognition motifs (RRM1–4), and RRM1-2 binds both the poly(A) tail and eIF4G component of eIF4F, resulting in enhancement of translation. Here, we show that PABP interacts with the 40S and 60S ribosomal subunits dynamically via RRM2-3 or RRM3-4. Using a reconstituted protein expression system, we demonstrate that wild-type PABP activates translation in a dose-dependent manner, while a PABP mutant that binds poly(A) RNA and eIF4G, but not the ribosome, fails to do so. From these results, functional significance of the interaction of PABP with the ribosome is discussed.

## Introduction

Most eukaryotic mRNA has a cap structure with m7GpppN (where N is any nucleotide) at the 5′ end and a poly(A) tail structure at the 3′ end. These terminal structures enhance translation. The cap structure is bound by eukaryotic translation initiation factor (eIF) 4F, which comprises three proteins (eIF4E, eIF4A and eIF4G). eIF4E interacts directly with the cap, and eIF4A is an ATP-dependent RNA helicase thought to work with the RNA-binding initiation factor eIF4B to unwind the secondary structure of the 5′-untranslated region of the mRNA. eIF4G is a large modular scaffolding protein with binding sites for eIF4E, eIF4A and eIF3, a multi-subunit initiation factor that interacts directly with the small (40S) ribosomal subunit^1,2^.

The poly(A) tail is bound by poly(A)-binding protein (PABP), which consists of four RNA-recognition motifs (RRMs; RRM1, 2, 3 and 4) and a C-terminal portion containing an unstructured region and an α-helical peptide-binding domain (PABC)^3^. The N-terminal two RRMs (RRM1-2) bind poly(A) RNA as well as eIF4G^4–6^.

Targeting of poly(A) RNA and eIF4G to RRM1-2 (specifically to RRM2 for eIF4G) of PABP is cooperative, ensuring communication between the mRNA 5′ and 3′ ends^7^. PABP binding to eIF4G activates translation initiation most probably by stabilizing the association of eIF4G with the mRNA^8,9^.

In addition, PABP harbors binding sites for other molecules important for translation and metabolism of RNA and proteins. Paip (PABP-interacting protein) 2 binds RRM2-3 of PABP in competition with the poly(A) RNA and eIF4G, resulting in translation inhibition^10,11^. Paip 2 also can bind PABC although less avidly, and a similar mode of binding to PABP is observed for Paip1^12^. Eukaryotic release factor 3 (eRF3) and the microRNA-related protein GW182 bind to PABC to influence translational and post-translational events^3,13^.

Here we report that PABP dynamically associates with the ribosomal RNAs, and this interaction might support translation.

## Materials and Methods

### Ribosomes

Ribosomes that were not depleted of PABP were purified as described^14^. PABP-free ribosomes were prepared as follows: a crude ribosome preparation obtained from HeLa S3 cells (1.5 L) was treated with 1 mM puromycin at 37 °C for 10 min and then centrifuged at 15,000 × *g* for 10 min at 4 °C. The supernatant was again centrifuged at 15,000 × *g* for 10 min at 4 °C. The supernatant (2.5 ml) was incubated with GST-Paip2^15^ (2 mg) at room temperature for 30 min and then resolved by the HiPrep 16/60 Sephacryl S-500 HR gel-filtration column (120 ml; GE Healthcare), equilibrated with a buffer (20 mM Tris-HCl pH 7.5, 150 mM KCl, 4 mM magnesium acetate, 6.8% (wt/vol) sucrose, 1 mM DTT). Elution was carried out with the same buffer at a flow rate of 0.5 ml/min, and 1.5-ml fractions were collected with the ÄKTAprime plus system (GE Healthcare). The fractions that contained ribosomes (fraction numbers: from 30 to 51) were combined and concentrated using the Amicon Ultra-15 (molecular weight cut-off 50,000) to approximately 5 ml.

This ribosome-rich sample was incubated again with GST-Paip2 (2 mg) at room temperature for 30 min. After incubation, the sample was mixed with KCl (final concentration, 500 mM) and a mixture of ATP and magnesium acetate (final concentration, 2 mM each) to disrupt contaminating chaperonin CCT^16^. The resulting mixture was resolved by 10–40% (wt/vol) sucrose-gradient centrifugation with a buffer (20 mM Tris-HCl pH 7.5, 500 mM KCl, 4 mM magnesium acetate, 2 mM DTT, 2 mM ATP-magnesium) at 25,000 rpm in the SW28 rotor (Beckman Coulter) for 16 h at 4 °C. Two-milliliter fractions were successively taken from the top of the gradient, and an aliquot of each fraction was analyzed by SDS-PAGE followed by Coomassie brilliant blue staining.

The fractions that mainly contained the 40S subunit were combined and diluted by four times with a sucrose-free buffer (20 mM Tris-HCl pH 7.5, 500 mM KCl, 4 mM magnesium acetate, 2 mM DTT). The diluted 40S fraction was concentrated using Amicon Ultra-15 (molecular weight cut-off 50,000) to approximately 5 ml. The fractions that mainly contained the 60S subunits were also diluted and concentrated in the same manner. These 40S and 60S fractions were incubated with GST-Paip2 (2 mg) at room temperature for 30 min and separately resolved by sucrose-gradient centrifugation in the same conditions as described above but without pre-treatment with ATP. The fractions that contained the 40S subunit only and the 60S subunit only were concentrated as described above and dialyzed against a 100-fold volume of a buffer (20 mM Tris-HCl pH 7.5, 100 mM potassium acetate, 4 mM magnesium acetate, 2 mM DTT) for 3 h at 4 °C using a dialysis membrane (molecular cut-off 50,000), and then against a new batch of the same buffer overnight.

The dialyzed samples of the 40S and 60S subunits were applied to GST-Paip2-bound Glutathione Sepharose 4B resin (0.2 ml, GE Healthcare). The unbound fraction was collected and then passed through Glutathione Sepharose 4B resin (0.2 ml) to remove possibly contaminating GST-Paip2. The samples were concentrated as described above and the concentration of 40S and 60S ribosomes was determined by measuring optical density (OD) at 260 nm^17^.

### Recombinant PABP and mutants

For PABP-His, PABP- His-PA, and deletion mutants-His-PA, a bacterial strain BL-21 (DE-3) was transformed with pET28b-PABP-His, pET28b-PABP-His-PA, or deletion mutants-His-PA and grown in Luria broth (300 ml) at 37 °C until OD at 600 nm reached 0.4 to 0.6. Isopropyl-β-D-thiogalactopyranoside was added to 0.1 mM, and cells were cultured at 30 °C for 12 to 16 h. After washing with a buffer (40 ml; 20 mM Tris-HCl pH 7.5, 150 mM NaCl), the bacterial pellet was kept at −20 °C until use. The frozen pellet was suspended in a buffer (20 ml; 20 mM HEPES pH 7.5, 2 M KCl, 10% glycerol, 5 mM β-mercaptoethanol, 1 mM EDTA, 0.1% Triton X-100, 20 mM imidazole, and 1 × protease inhibitor cocktail (EDTA-free; Nacalai)), lysed by sonication, and centrifuged at 15,000 × *g* for 30 min at 4 °C. The supernatant was then loaded onto a Ni-NTA resin (1 ml, Qiagen), and the resin was washed with the same buffer (10 ml) and further washed with another buffer (10 ml; 20 mM HEPES, pH 7.5, 100 mM KCl, 10% glycerol, 5 mM β-mercaptoethanol, 1 mM EDTA). The proteins were eluted in a stepwise manner with increasing concentrations of imidazole (50, 100, 250, 500, and 1000 mM) in a buffer (5 ml each; 20 mM HEPES-KOH pH 7.5, 100 mM KCl, 10% glycerol, 5 mM β-mercaptoethanol, 1 mM EDTA). The eluates with 50, 100, and 250 mM imidazole were combined and passed through the Q Sepharose resin (1 ml, GE Healthcare) to remove possibly contaminating RNA. The flow-through fraction was applied onto the Heparin Sepharose resin (1 ml, GE Healthcare), and the resin was then washed with a buffer (10 ml; 20 mM HEPES-KOH pH 7.5, 100 mM KCl, 10% glycerol, 5 mM β-mercaptoethanol, 1 mM EDTA). Bound proteins were eluted in a step-wise manner that increased the concentration of KCl from 300 to 1000 mM in a buffer (5 ml each; 20 mM HEPES-KOH pH 7.5, 10% glycerol, 5 mM β-mercaptoethanol, 1 mM EDTA). The eluates with 300 and 400 mM KCl were combined and concentrated by Amicon Ultra-15 (molecular cut-off 10,000) to approximately 2.5 ml and then loaded onto a PD-10 column (GE Healthcare) equilibrated with a buffer (20 mM HEPES-KOH pH 7.5, 100 mM KCl, 10% glycerol). After elution with 3.5 ml of the same buffer, the eluate was concentrated.

PABP- or 12C-FLAG: BL-21 (DE3) was transformed with pET28 PABP-FLAG or pET28 12C-FLAG. Proteins were expressed as described for PABP-His-PA and purified with anti-FLAG M2 agarose chromatography (Sigma).

### Binding assay using sucrose-gradient centrifugation

PABP-His (60 pmol) was mixed with the 40S subunit (30 pmol) or the 60S subunit (30 pmol) in a buffer (200 μl; 20 mM Tris-HCl pH7.5, 0.1 M KCl, 4 mM magnesium acetate, 2 mM DTT) in the presence or absence of an RNA fragment (Supplementary Table S1) (60 pmol) at 4 °C for 15 min. The mixture was then resolved by 10–40% sucrose-gradient centrifugation in the same buffer at 38,000 rpm in the SW41Ti rotor (Beckman Coulter) for 3 h at 4 °C. Fractions of 1 ml were successively taken from the top of the gradient, and 15 μl of each fraction was analyzed by SDS-PAGE followed by western blotting with anti-PABP (ab21060, Abcam), anti-S6 (#2317, Cell Signaling Technology) or anti-L13a (#2765, Cell Signaling Technology) antibodies. Another 15 μl of each fraction was analyzed by denaturing 1% agarose gel electrophoresis followed by northern blotting with a corresponding antisense probe labeled with digoxigenin-UTP (Roche).

### *In vitro* binding assay

Full-length PABP-His-PA or truncated versions of the protein (3 μg each) were immobilized on TALON metal affinity resin (10 μl, TaKaRa) by incubation in a binding buffer (100 μl; 20 mM HEPES-KOH pH 7.5, 100 mM KCl, 10% glycerol, 4 mM magnesium acetate, 0.1% Triton X-100, 1 mg/ml bovine serum albumin) at room temperature for 30 min. After incubation, the unbound fraction was removed by centrifugation at 5000 × *g* for 30 s at 4 °C. The resin was washed three times with a washing buffer (100 μl each; 20 mM HEPES-KOH, pH 7.5, 100 mM KCl, 10% glycerol, 4 mM magnesium acetate, 0.1% Triton X-100). Then, the 40S subunit or the 60S subunit (4.8 pmol each) was added to the resin in the binding buffer (50 μl) and incubated on ice for 15 min. After incubation, the unbound fraction was removed and the resin was washed five times with the washing buffer (400 μl each). Bound materials were eluted by incubation with a buffer (15 μl; 20 mM HEPES-KOH pH 7.5, 100 mM KCl, 10% glycerol, 250 mM imidazole) for 10 min at room temperature. The eluate was analyzed by western blotting with antibodies against ribosomal protein S6, ribosomal protein L13a or the PA tag (WAKO).

### Reconstituted translation system

The following components were mixed in a test tube: eIF1 (61 ng), eIF1A (76 ng), eIF2 (547 ng), eIF2B (51 ng), eIF3 (100 ng), eIF4A (570 ng), eIF4B (144 ng), eIF4G/eIF4E (141 ng), eIF5 (76 ng), eIF5B (85 ng), DHX29 (67 ng), tRNA (5 μg), eEF1 (25 μg), eEF2 (500 ng), eRF1 (125 ng), eRF3 (125 ng), 40S ribosomal subunit (4.8 pmol), 60S ribosomal subunit (4.8 pmol), aminoacyl-tRNA synthetases (750 ng), amino acid mixture (100 μM, Promega), and 10 × reaction buffer (0.5 μl; 500 mM HEPES-KOH, pH 7.5, 552 mM potassium acetate, 60 mM magnesium acetate, 2 mM spermidine trihydrochloride, 0.6 mg/ml creatine kinase, 206 mM creatine phosphate, 10 mM DTT, 13.6 mM ATP, and 8.4 mM each of GTP, UTP, and CTP). This combination (4.5 μl) was then mixed with a template RNA (0.5 μl) and PABP or a truncated PABP protein (0.5 μl; the final concentration is described in each figure), and incubated for 1.5–6 h at 32 °C.

### Analysis of *in vitro* translated products

An aliquot (1 μl) from the translated sample was used for the luciferase reporter assay using the Dual-Luciferase Reporter Assay system (Promega). The remaining sample (4 μl) was subjected to western blotting with anti-HA tag (16B12, BioLegend) or anti-Myc tag (9B11, Cell Signaling Technology) antibody. Protein bands were analyzed using the ImageQuant LAS 4000 mini (Fujifilm).

For the *in vitro* dissociation experiment, CRAC analysis and other methods, see Supplementary Methods.

## Results

### Binding of PABP to the ribosome

**Figure 1:**
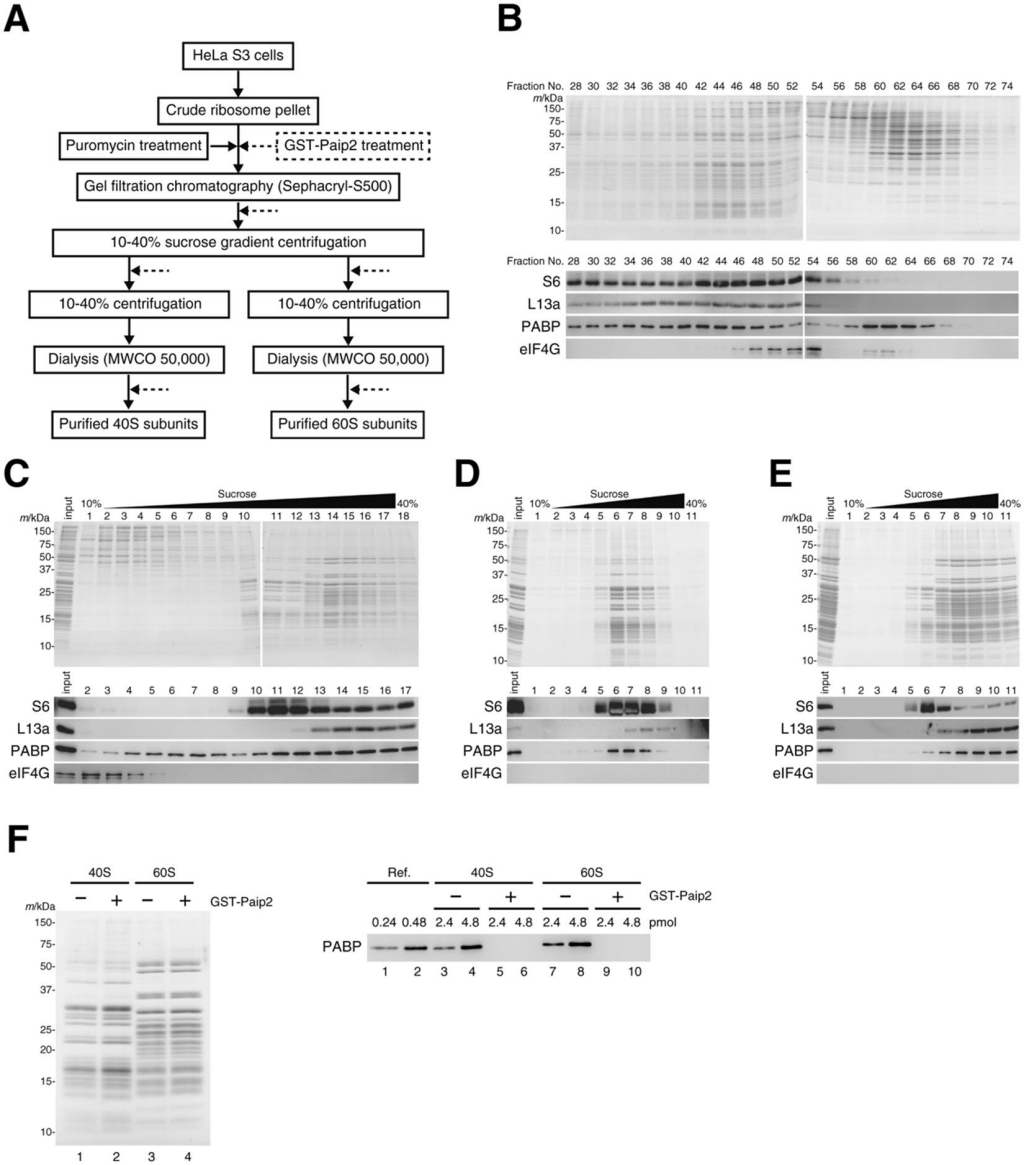
Co-purification of PABP with the ribosome. (**A**) Diagram of purification procedure of the 40S and 60S ribosomal subunits from HeLa cells. (**B**) Gel filtration chromatography of the puromycin-treated ribosomal sample. (**C**) First sucrose-gradient centrifugation. (**D**) Second sucrose-gradient centrifugation for purification of the 40S subunit. (**E**) Second sucrose-gradient centrifugation for purification of the 60S subunit. (**F**) Purified 40S and 60S subunits. Left panel: Coomassie brilliant blue-staining of the 40S and 60S subunits (2.4 pmol, each) purified with (lanes 2, 4) or without (lanes 1, 3) GST-Paip2 treatments. Right panel: western blot of the purified 40S and 60S subunits (2.4 or 4.8 pmol) for PABP (lanes 3–10). PABP-His (lane 1, 0.24 pmol; lane 2, 0.48 pmol) was loaded as the reference.

During purification of ribosomes from HeLa cells according to the procedure outlined in Fig. 1A, we found that PABP co-purified with ribosomes as described below.

A crude ribosomal preparation from HeLa cell lysates was treated with puromycin and resolved by gel-filtration chromatography (Fig. 1B). Each fraction was assessed using SDS-PAGE followed by Coomassie brilliant blue staining (Fig. 1B, upper panel) and western blot with anti-S6 (for the 40S subunit), anti-L13a (for the 60S subunit), anti-PABP, and anti-eIF4G (as a reference) antibodies (Fig. 1B, lower panel). Ribosomes were mainly found in fractions 28 to 54 (Fig. 1B), in which PABP also eluted; in addition, PABP eluted in fractions 58 to 66, probably representing the PABP monomer and/or RNA-bound multimers of PABP.

These fractions containing both the ribosomes and PABP were resolved by sucrose- gradient centrifugation (Fig. 1C). The 40S and 60S subunits were found mainly in fractions 10 to 12 (the 40S fractions) and 13 to 17 (the 60S fractions), respectively. Of importance, these fractions contained PABP.

To further purify 40S and 60S ribosomal subunits, these 40S and 60S fractions were individually subjected to the second sucrose-gradient centrifugation (Fig. 1D,E, respectively). The 40S subunits and PABP co-migrated around fractions 6 to 8 (Fig. 1D), and the 60S subunits and PABP co-migrated around fractions 8 to 11 (Fig. 1E). Thus, PABP is likely to associate with ribosomes; indeed, binding of PABP to ribosomes in yeast has been reported^18^, and recent proteomics studies included PABP in the list of the numerous ribosome-associated proteins of yeast^19^ and mammals^20^.

The purified 40S and 60S ribosomal subunits were estimated to contain PABP molecules roughly at a 10:1 molar ratio each, meaning that the molar ratio of PABP to the ribosome (40S + 60S subunits) would be 1:5 in the final preparation, as assayed by western blot (Fig. 1F). Because PABP is dissociated from the ribosome during purification, more PABP molecules would be associated with the 40S and 60S subunits in cells. To estimate how many PABP molecules would bind to the ribosome before purification, the puromycin-treated crude ribosomal preparation was resolved by the sucrose-gradient centrifugation. The fractions containing the ribosome were combined for western blot. The molar ratio of PABP to the ribosome (40S + 60S subunits) was roughly 2:1 (Supplementary Fig. S1). Thus, it could be roughly estimated that at least two PABP molecules would associate with one ribosome in cells.

To study the functional significance of PABP association with the ribosome, we first sought to obtain PABP-free ribosomes. To this end, we treated the ribosomal samples with glutathione *S*-transferase (GST)-Paip2^15^ at multiple purification steps, indicated by dotted arrows in Fig. 1A, as cell extracts can be cleared of PABP by passing through GST-Paip2-affinity chromatography^15^. With this procedure, both the 40 and 60S subunits were depleted of PABP to undetectable levels (Fig. 1F).

### Dynamic interaction of PABP with the ribosome

**Figure 2:**
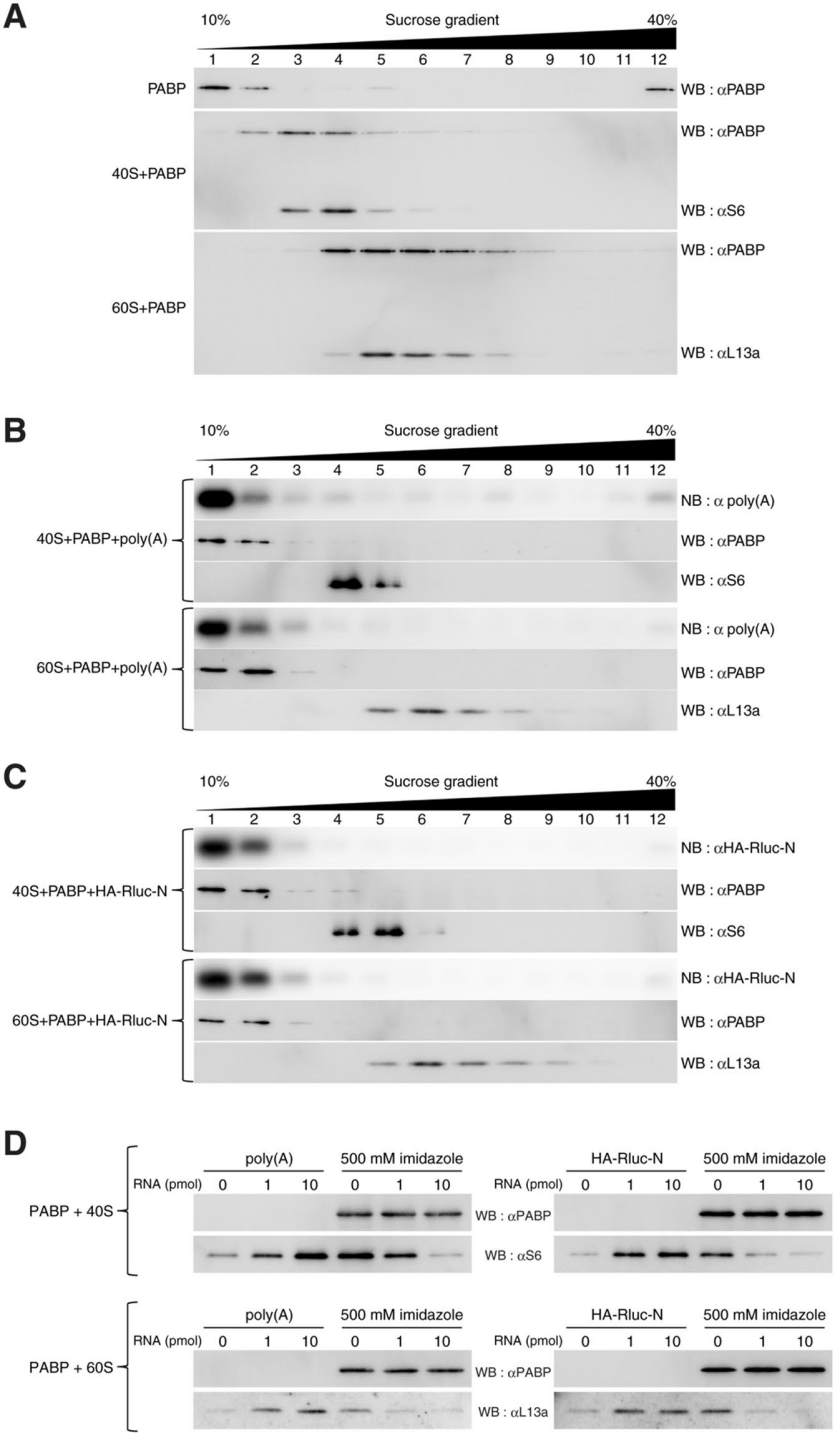
Dynamic interaction of PABP with ribosomes. (**A**–**C**) Fractions after sucrose-gradient centrifugation were analyzed by western blotting (WB: PABP, 40S and 60S) or northern blotting (NB: RNA). (**A**) PABP (60 pmol) alone (top panel) or a mixture of PABP (60 pmol) and the 40S subunit (30 pmol) (middle panel) or the 60S subunit (30 pmol) (bottom panel) was resolved by sucrose-gradient centrifugation. (**B**) A mixture of PABP (60 pmol), the poly(A) RNA (60 pmol), and the 40S subunit (30 pmol) or the 60S subunit (30 pmol) was resolved by sucrose-gradient centrifugation. (**C**) A mixture of PABP (60 pmol), the HA-Rluc-N RNA (60 pmol), and the 40S subunit (30 pmol) or the 60S subunit (30 pmol) was resolved by sucrose-gradient centrifugation. (**D**) Incubation with the poly(A) RNA (left panels) or HA-Rluc-N RNA (right panels) dissociated the 40S (upper panels) or 60S subunit (lower panels) from the PABP-His complex immobilized on the TALON resin (left three lanes, each panel). After incubation with RNA followed by washing, RNA/proteins were eluted with imidazole (500 mM) from the resin (right three lanes, each panel).

To confirm that PABP can associate with ribosomes, we mixed the PABP-depleted ribosomes with a recombinant PABP protein (PABP-His) and resolved the mixture by 10–40% sucrose-gradient centrifugation. When PABP alone was resolved, it mainly remained near the top of the gradient (Fig. 2A, top panel). In contrast, when mixed with the 40S subunit, PABP sedimented at heavier fractions (fractions 3 and 4) where the 40S subunits were also fractionated (Fig. 2A, middle panel and Supplementary Fig. S2A). In addition, when PABP was mixed with the 60S subunit, a large portion of the PABP molecules co-sedimented with the 60S subunits (Fig. 2A, bottom panel and Supplementary Fig. S2A); gradual dissociation of PABP from the ribosomal subunits may account for the distribution of PABP in lighter fractions in both cases. Thus, we conclude that PABP interacts with the 40S and 60S ribosomal subunits.

Because PABP binds poly(A) RNA, we next examined whether a ternary complex consisting of the ribosome, PABP and poly(A) RNA could be formed. When poly(A) RNA (Supplementary Table S1), PABP and the 40S or 60S subunit were mixed and resolved by sucrose-gradient centrifugation, the poly(A) RNA did not co-sediment with the ribosome (Fig. 2B and Supplementary Fig. S2B). Unexpectedly, PABP did not co-migrate with the ribosome in the presence of the poly(A) RNA (Fig. 2B). To examine whether the separation of PABP from the ribosomal subunits was specifically because of the poly(A) RNA, we performed a similar experiment with a non-poly(A) RNA (HA-Rluc-N: RNA sequence encoding an N-terminal portion of the HA-Rluc mRNA) (Supplementary Table S1) and found that PABP was also separated from the ribosome in the presence of this RNA (Fig. 2C). These results suggest that RNA-free PABP molecules interact with the ribosome and can be dissociated from the ribosome by RNA.

To confirm that RNA dissociates PABP from the PABP–ribosome complexes, we immobilized PABP-His on the TALON affinity resin, followed by incubation with the PABP-free 40S or 60S subunit. After a wash, the resin was incubated with an increasing amount of the poly(A) RNA or the HA-Rluc-N RNA. The 40S and 60S subunits were dissociated from the resin in an RNA dose-dependent manner, leaving PABP-His on the resin (Fig. 2D). These results suggest that PABP interacts with the ribosome and RNA in a dynamic manner.

Because PABP interacts with eIF4G, we then examined whether a ternary complex consisting of the ribosome, PABP, and eIF4G could be formed. When eIF4G/4E, PABP, and the 40S or 60S subunit were mixed and resolved by the sucrose-gradient centrifugation, eIF4G did not co-sediment with the ribosomes (Supplementary Fig. S2C), consistent with the sucrose-gradient centrifugation results shown above (Fig. 1C). Thus, it is unlikely that PABP binds both eIF4G and the ribosome simultaneously.

### The ribosome binds to RRM2-3 and 3-4 of PABP

As noted, PABP contains four N-terminal RRM (1–4) domains and the C-terminal domain (PABC, abbreviated as C in the following deletion mutants). Poly(A) RNA binds to RRM1-2, and eIF4G binds to RRM2^7^, while the translation repressor Paip2 mainly binds to RRM2-3^10^. In addition, PABC harbors the binding sites for eRF3 and other molecules^3^.

**Figure 3:**
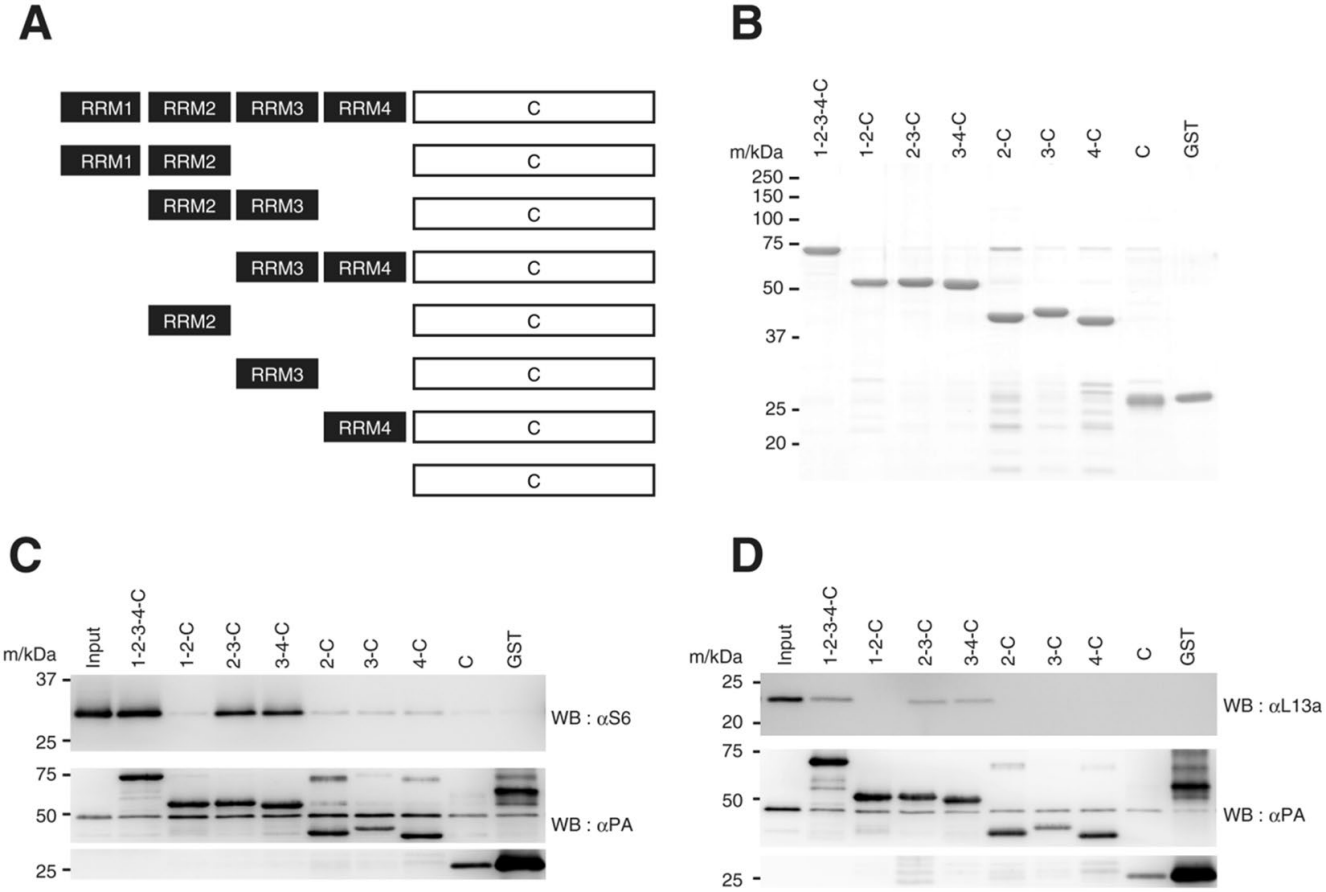
Localization of ribosomes on PABP. (**A**) A schematic representation of PABP and RRM-deletion mutants. (**B**) C-terminally His-PA–tagged wild-type PABP (1-2-3-4-C), RRM-deletion mutants and GST proteins (1 μg each, Coomassie brilliant blue stained). (**C**) and (**D**) Binding of the 40S ribosomal subunit (**C**) and the 60S subunit (**D**) to PABP (1-2-3-4-C) or its deletion mutants immobilized on the TALON resin. Input: one twentieth of the ribosomal sample was analyzed at the same time.

To determine which portion of PABP is required for ribosomal binding, we carried out an *in vitro* binding assay using recombinant PABP fragments and the PABP-free 40S or 60S ribosomal subunits. The carboxy-terminal His-PA-tagged PABP protein (1-2-3-4-C-His-PA), various RRM-deletion mutants (Fig. 3A), and GST-His-PA (a negative control) were expressed in bacteria and purified (Fig. 3B). These proteins were immobilized on the TALON affinity resin and incubated with the PABP-free 40S subunit or 60S subunit. After a wash, elution was performed with imidazole. The eluates were resolved by SDS-PAGE and analyzed by western blotting with anti-PA (for PABP, deletion mutants and GST), anti-S6 (for 40S), or anti-L13a (for 60S) antibodies (Fig. 3C,D). The 40S subunit interacted with 2-3-C and 3-4-C as well as with full-size PABP (1-2-3-4-C), but not with GST, 1-2-C or C. Because 2 C, 3 C, and 4 C respectively failed to retain the 40S subunit, the site of the 40S ribosome binding is likely to span the two RRMs (RRM2-3 or RRM3-4) (Fig. 3C). A similar binding profile was observed for the 60S binding with PABP, although the binding strength of this subunit to PABP was apparently weaker than that of the 40S subunit (Fig. 3D, compared with 3C). This putative weak binding of the 60S subunit to PABP might arise from steric hindrance by the resin, as the binding assay using sucrose-gradient centrifugation showed no clear difference between the two subunits in binding activity to PABP (Fig. 2).

### Localization of the PABP-binding sites on the ribosome

**Figure 4:**
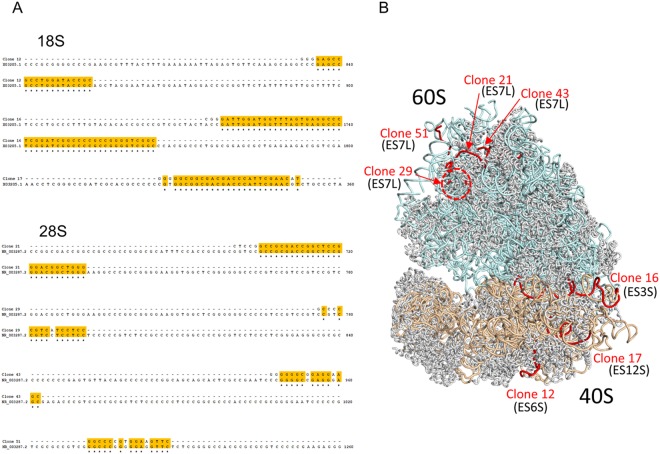
PABP binds to expansion segments of the mammalian ribosome. (**A**) Alignment of the PABP-cross-linked RNA sequences to human 18S (GenBank: X03205.1) and 28S (NR_003287.2) ribosomal RNAs. (**B**) PABP binding sites in the structure of the human ribosome (PDB ID: 4UG0)^40^. Ribbon structures of the ribosomal proteins, 60S-subunit rRNAs (28S, 5.8S, and 5S), and 40S-subunit rRNA (18S), colored gray, cyan, and wheat, respectively, are represented from the A-site side. The PABP cross-linked rRNA sites are indicated by red. The site corresponding to clone 29 is localized in the disordered region, whose root is indicated by the red dotted circle. ES: expansion segment^23^.

We next determined the PABP-binding sites on the ribosome using the CRAC (ultraviolet (UV)-cross-linking and analysis of cDNAs) method^21,22^ with some modifications (Supplementary Methods). The PABP-depleted 40S or 60S subunits and the recombinant PABP-His-PA protein were incubated and irradiated with UV light. The samples were then treated with guanidine-HCl to disrupt the ribosomal structure and incubated with a nickel resin. After extensive washes, the resin was treated with RNases, and protein–RNA complexes containing PABP-His-PA were eluted with imidazole. Complementary DNA for the UV-crosslinked RNA was cloned and sequenced (Supplementary Fig. S3). The sequences of these cDNAs were assigned to the expansion segments of the 40S (ES3S, ES6S and ES12S) and 60S subunits (ES7L)^23^ (Fig. 4). Of note, some of the sequences do not match the reported rRNA sequences perfectly (Fig. 4A), probably because of errors in reverse transcription at the nucleotides that are cross-linked^21^. The expansion segments are eukaryote-specific RNA segments protruding from the core of the ribosome^23^. However, the possibility is not excluded that PABP interacts with other sequences of the ribosomal RNA and/or ribosomal proteins in addition to the expansion segments.

### Establishing a reconstituted cap/poly(A)-supported protein synthesis system

To gain insight into the functional significance of PABP-association with the ribosome, we established a cap/poly(A)-supported protein synthesis system reconstituted with mammalian (human) factors. We previously constructed an eIF-independent translation system by assembling the 40S subunit, 60S subunit, eEF1, eEF2, eRF1/3, tRNAs and aminoacyl tRNA synthetases with an HCV (hepatitis C virus) IRES (internal ribosome entry site)-driven template^14^. To establish an eIF-dependent translation system, we purified all the eIFs (Supplementary Fig. S4) and added them to the eIF-independent translation system. The complete system, which contained all of the eIFs, and the systems that respectively lacked an individual eIF were programmed with a capped and poly(A)-tailed Renilla luciferase (Rluc) RNA (cap-Rluc-A), and Rluc activity was assayed to assess dependency of this system on each eIF. This system was found to be highly dependent on eIF4F (eIF4E, eIF4A, and eIF4G), eIF4B and DHX29 as well as on eIF1A and eIF2 (Supplementary Fig. S5).

**Figure 5:**
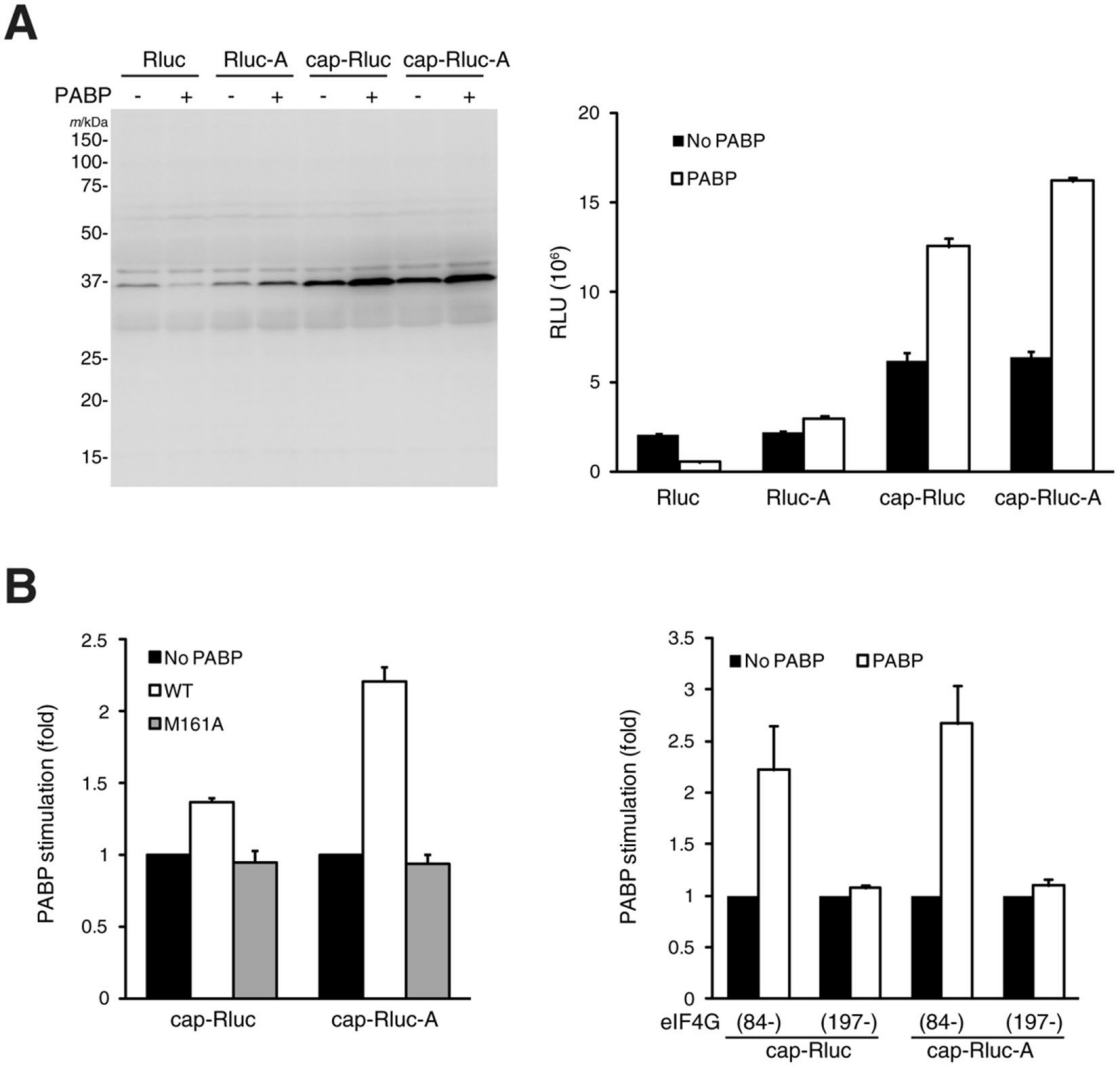
Stimulation of translation by PABP in the reconstituted cap-dependent translation system. (**A**) Rluc, Rluc-A, Cap-Rluc or Cap-Rluc-A RNA (0.1 μM each) was translated in the reconstitution system with or without PABP (1.92 μM). After translation, samples were analyzed by western blotting with an anti-myc antibody (left panel) and Rluc assay (right panel; each column and bar represent the mean and standard deviation of three experiments, respectively). eIF4G (84–1599), which contains the PABP binding site^24^, was used as eIF4G. (**B**) Cap-Rluc or Cap-Rluc-A (0.1 μM each) RNA was translated in the reconstitution system with or without PABP or a PABP mutant (M161A)^9^ (1.92 μM, each) (left panel). These RNAs were also translated with eIF4G (84–1599) or eIF4G (197–1599)^24^ in the presence or absence of PABP (1.92 μM) (right panel). After translation, Rluc activity was measured. Each column and bar represent the mean and standard deviation three experiments, respectively; the average Rluc activity without PABP was set at 1.0.

We then incubated this system with each of the four types of Rluc-based mRNA (Rluc: uncapped without poly(A); Rluc-A: uncapped with poly(A); cap-Rluc: capped without poly(A); and cap-Rluc-A: capped with poly(A); the open reading frame was tagged with HA and myc at the N- and C-termini, respectively) in the presence or absence of PABP. After incubation, translational activity was assessed by western blot with an anti-myc antibody and by Rluc activity. Both assays showed that the cap-structure and poly(A) tail stimulate translation, and the highest productivity was observed with the capped and poly(A)-tailed RNA in the presence of PABP (Fig. 5A).

When a PABP mutant (M161A)^9^, which cannot bind to eIF4G, was used in place of wild-type PABP, no activation was observed regardless of the presence of the poly(A) tail (Fig. 5B, left panel). Furthermore, when an N-terminally truncated eIF4G (197–1599)^24^, which does not contain the PABP binding site, was used in place of eIF4G (84–1599), stimulation by PABP was abolished (Fig. 5B, right panel). These data are consistent with the well-supported concept that binding of eIF4G to PABP is crucial for functional communication between the 5′ and 3′ ends of mRNAs in translation^5,8,9,25^, and validate the usefulness of this system for studies of translational control by the cap and poly(A) structures.

### Functional analyses using the reconstituted translation system

**Figure 6:**
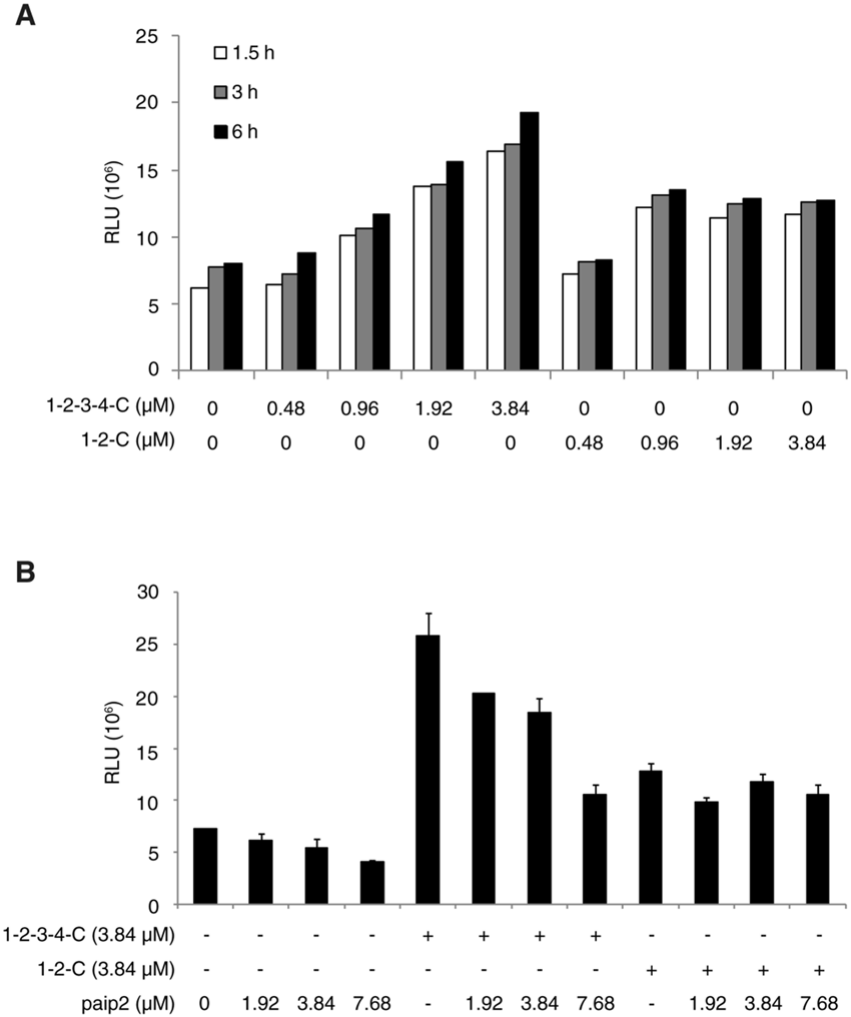
Functional analysis of PABP interaction with the ribosome. (**A**) Cap-Rluc-A RNA (0.1 μM) was translated in the reconstitution system in the presence of increasing concentrations (0 to 3.84 μM) of PABP (1-2-3-4-C) or a truncated PABP (1-2-C). At indicated times, an aliquot of each sample was removed for the Rluc assay. Each bar represents the mean of two experiments. (**B**) Cap-Rluc-A RNA (0.1 μM) was translated in the reconstitution system with PABP or PABP (1-2-C) (3.84 μM each) in the presence of increasing concentrations (0 to 7.68 μM) of Paip2 for 6 h. After translation, Rluc activity was measured. Each column and bar represent the mean and standard deviation of three experiments, respectively.

Having established the reconstituted cap/poly(A)-supported translation system, we explored the functional significance of the association of PABP with the ribosome. For this aim, we used PABP (1-2-C), a PABP mutant that binds to poly(A) and eIF4G^7^, but not to the ribosome (Fig. 3). Low doses of PABP and PABP (1-2-C) supported translation of the cap-Rluc-A RNA similarly. At higher doses, in contrast, activation of translation by PABP (1-2-C) plateaued, while PABP continued to enhance translation in a dose-dependent manner (Fig. 6A). A similar profile was obtained with the cap-Rluc RNA except that the highest dose (3.84 μM) of PABP and PABP (1-2-C) inhibited translation considerably (Supplementary Fig. S6).

An increasing amount of Paip2 stoichiometrically repressed activation by PABP (Fig. 6B), confirming that Paip2 is an important regulator of translation^26,27^. The translation repression by Paip2 might in part trace to dissociation of PABP from the ribosome (Fig. 1). In contrast, Paip2 exerted little effect on translation supported by PABP(1-2-C) (Fig. 6B), probably because Paip2 may not bind to PABP(1-2-C) as strongly as to PABP; Paip2 mainly binds to RRM2-3 of PABP^12^.

Because PABP has been considered to play a role in recycling of ribosomes, we conducted experiments with PABP and PABP(1-2-C) in the presence of ABCE1^28^, a eukaryotic recycling factor. The protein synthesis yield was enhanced by ABCE1, as expected (Supplementary Fig. S7A), but the translational profile concerning the effects of the full-length PABP and PABP (1-2-C) was comparable to that obtained from the experiments without ABCE1 (compare Supplementary Figs S7B and 6A).

These outcomes might mean that, although PABP in general binds directly to mRNA and may thus stimulate translation, some of the highly concentrated PABP molecules could be trapped by the ribosome for translation activation; a large part of the same concentration of PABP (1-2-C) molecules would remain unavailable to translation. However, the possibility is not rigorously excluded that RRM3-4 would have a function other than binding to the ribosome, and truncation of RRM3-4 would diminish the PABP function in translation.

## Discussion

Here, we demonstrate that the ribosome dynamically interacts with PABP. PABP is a quite abundant protein^29^, with an estimated concentration of 4 μM in HeLa cells^17^. Consequently, only 30% of PABP molecules are thought to bind to the poly(A) tail of mRNAs with the majority of PABP being associated with non-poly(A) RNAs^29^. Considering that the concentration of ribosomes is also high (approximately 1.5 μM in HeLa cells)^17,29^, the ribosome would be expected to be a primary site for free PABP to bind. Because the interaction between PABP and the ribosome is dynamic; PABP is readily dissociated with a non-specific RNA (Fig. 2), PABP molecules trapped transiently by the ribosome would be transferred to the nearest mRNA, most probably to the translated mRNA, and then to eIF4G. An excess amount of PABP is also titrated by Paip2^26,27,30^ and Paip2 dissociates PABP from the ribosome (Fig. 1). Thus, it is conceivable that PABP molecules in cells dynamically associate with and dissociate from mRNAs, the ribosome, eIF4G and Paip2.

We have established a cap-dependent protein synthesis system reconstituted with purified mammalian components. Using this system, we confirm that the interaction of PABP with eIF4G is crucial for PABP-mediated translational activation (Fig. 5). Nonetheless, it has been suggested that PABP also plays a role in 60S subunit joining^9,31^, probably not via eIF4G. Binding of PABP to the 60S subunit might account for this role, although the mechanism remains to be explored.

PABP primarily binds the poly(A) tail, as its name indicates, but it also is reported to bind RNA sequences other than the poly(A) tails. PABP binds to AU-rich sequences in the 5′ and 3′ untranslated regions^32^, including the poly(A) signal^33^ and A-rich sequences of non-coding RNAs, probably via RRM1-2 and/or RRM3-4^34^. The expansion segments of the ribosome, which are GC-rich^23^, likely interact with RRM2-3 and RRM 3-4 of PABP (Figs 3 and 4). These two combinations of PABP RRM (2-3 and 3-4) were previously shown to have higher affinity for poly(G) than poly(A) RNA^4^. However, because PABP is dissociated from the ribosome with a non-specific RNA (Fig. 2), the interaction of the expansion segments with RRM2-3 and RRM3-4 likely is not sequence-specific. Of note, Xenopus RRM3-4 of PABP tethered to the 3′ untranslated region of the mRNA stimulates translation^35^, suggesting the involvement of RRM3-4 in translation.

In the present (Fig. 5) and previous^9^ experiments, PABP also was shown to stimulate translation of poly(A)-minus mRNA to some extent. It is likely that PABP is recruited to the translation machinery in multiple ways, including through the ribosome. For example, RNA-binding proteins such as the herpes simplex virus-1 protein, ICP27 and a cellular mRNA-specific regulator, Deleted in Azoospemia-like (called Dazl) bind both PABP and a specific mRNA to enhance its translation^36,37^. Of note, the length of the poly(A) tail is not directly related to translational efficiency in somatic cells^38,39^.

Finally, we demonstrated the PABP-binding sites included the expansion segments of the ribosome. The expansion segments of the mammalian ribosome consist mostly of duplexed RNA, and some of the segments protrude from the ribosome body like tentacles^23^. The expansion segments might be used as hubs for PABP because the segments that protrude from the ribosomal body are unlikely to participate in protein synthesis directly; therefore, the binding to them should not interfere with translation. Thus, these segments might trap not only PABP but also other RNA-binding proteins to support translation.

## Electronic supplementary material

Supplementary information

## Data Availability

Unprocessed images in each Figure are available at Supplementary Information.
