# Supplementary material for: Dynamic interaction of poly(A)-binding protein with the ribosome
Source: Sci Rep. 2018 Nov 28;8:17435. doi: 10.1038/s41598-018-35753-1 (PMC6261967; doi:10.1038/s41598-018-35753-1)

Supplementary Information

Dynamic interaction of poly(A)-binding protein with the ribosome

Kodai Machida, Tomoaki Shigeta, Yuki Yamamoto, Takuhiro Ito, Yuri Svitkin, Nahum Sonenberg and Hiroaki Imataka

Contents:

Supplementary Methods
Supplementary References
Supplementary Figures (S1, S2, S3, S4, S5, S6 and S7)
Supplementary Table
Unprocessed images for each Figure

Supplementary Methods

Plasmids

The plasmids pET28 PABP-His-PA and pET28 PABP-FLAG were constructed by polymerase chain reaction (PCR) using pET28 PABP-His as the template [1]; the amino acid sequence of the PA tag is GVAMPGAEDDVV (WAKO, Japan). For expression of RRM-deletion mutants, PCR-amplified DNA fragments encoding RRM2, RRM3, RRM4, RRM1-2, RRM2-3 or RRM 3-4 [2], were incorporated along with the PABC-encoding fragment [2] tagged His-PA or FLAG into the vector pET28b (Novagen).

The plasmid pUC-T7-HA-Rluc-myc-polyA$_{118}$ was constructed by replacing the Firefly luciferase coding region with the DNA fragment encoding HA-*Renilla* luciferase-myc in the plasmid pUC-T7-luc-polyA [3].

Proteins

Recombinant eIF1, eIF1A, eIF4E, eIF4A, eIF4B and eIF5: hexahistidine (His)-tagged eIF1, His-eIF1A, non-tagged eIF4E, non-tagged eIF4A, FLAG-eIF4B, His-eIF5, and non-tagged 4EBP1 were obtained as previously described [4].

Recombinant Paip2: GST-Paip2 was expressed and purified as described [5].

Recombinant eRFs and and native eEF2: non-tagged eRF1, non-tagged eRF3, and eEF2 were obtained as previously described [6].

Native eIF2: eIF2 was purified from HeLa cells as follows: a crude ribosomal pellet (0.8 g) prepared from HeLa S3 cells (5 l) [6] was suspended in a buffer (8 ml; 20 mM HEPES-KOH pH 7.5, 50 mM KCl, 5 mM magnesium acetate, 1 mM DTT, 250 mM sucrose) with stirring at 4°C. After addition of KCl to the suspension to the final concentration of 0.5 M, the mixture was further stirred for 1 h and centrifuged at 38,000 rpm in an SW41 rotor (Beckman Coulter) for 16 h at 4°C. The supernatant was mixed with ammonium sulfate to 40% saturation and centrifuged at 15,000 ×*g* for 30 min at 4°C. The resulting precipitate was stored at -85°C until use for eIF3 purification. The supernatant was brought to 50% saturation in ammonium sulfate, and precipitates were collected by centrifugation. The pellet was dissolved in buffer A (2.5 ml; 20 mM HEPES-KOH pH 7.5, 10% glycerol, 1 mM DTT, 0.5 mM EDTA) containing 100 mM KCl and loaded onto a PD-10 column (GE Healthcare) equilibrated with the same buffer. The eluate (3.5 ml) was applied onto Q Sepharose chromatography (0.4 ml, GE Healthcare). After washing with the same buffer (4 ml), eIF2-containing fraction was eluted by buffer A (2 ml) containing 400 mM KCl. The eluate was diluted by 1.5-times with buffer A and then loaded onto P11 phosphocellulose resin (0.4 ml, Whatmann) equilibrated with buffer A containing 300 mM KCl. The resin was washed with the same buffer (4 ml) and

incubated with buffer A (2 ml) containing 500 mM KCl to elute out eIF2 proteins. The eluate was applied to a PD-10 desalting column equilibrated with 20 mM HEPES-KOH pH 7.5, 100 mM KCl, 10% glycerol followed by concentration using an Amicon Ultra-15 device (molecular cut-off 30,000; Merck Millipore). After quantification of purified eIF2 by Bradford assay, the product was snap-frozen in liquid nitrogen and stored at -85°C. The eIF2-containing fraction was analyzed by western blotting with anti-eIF2α antibody (Invitrogen) and anti-eIF2β antibody (SantaCruz).

Recombinant eIF2B: BHK-21 cells grown to confluence on thirty 150-mm dishes were infected with a T7 RNA polymerase-expressing vaccinia virus (LO-T7-1), and then transfected with five plasmids: pUC-T7-EMCV-eIF2Bα, pUC-T7-EMCV-eIF2β, pUC-T7-EMCV-His-eIF2Bγ, pUC-T7-EMCV-His-eIF2Bδ, and pUC-T7-EMCV-His-eIF2Bε [4] as described [7]. After 48 h, cells were harvested and stored at -85°C. The frozen cells were lysed in a buffer (20 ml; 20 mM HEPES-KOH pH 7.5, 100 mM KCl, 10% glycerol, 0.1% Triton X-100, 2 mM β-mercaptoethanol, 1 mM EDTA, 10 mM imidazole). After centrifugation at 15,000 ×*g* for 30 min at 4°C, the supernatant was loaded onto a Ni-NTA resin (1 ml, Qiagen). The resin was washed with buffer B (10 ml; 20 mM HEPES-KOH pH 7.5, 10% glycerol, 2 mM β-mercaptoethanol, 1 mM EDTA) with 500 mM KCl and 10 mM imidazole, and further washed with buffer B (10 ml) with 100 mM KCl and 10 mM imidazole. His-eIF2B was eluted in a stepwise manner with increasing concentrations of imidazole (50, 100, 250, 500, and 1000 mM) in buffer B (5 ml, each). The eluates with 100, 250, 500, and 1000 mM imidazole were combined, and applied to a PD-10 desalting column equilibrated with 20 mM HEPES-KOH pH 7.5, 100 mM KCl, 10% glycerol to exchange the buffer. The sample was finally concentrated with Amicon Ultra-15 (molecular cut-off 50,000).

Native eIF3: The precipitates from the salt-washed fraction of the crude ribosome with ammonium sulfate (40% saturation; see the "eIF2" section) was suspended in buffer C (4 ml; 20 mM HEPES-KOH pH 7.5, 10% glycerol, 1 mM DTT, 0.1 mM EDTA) with 100 mM KCl. The suspension was dialyzed against a 100-times volume of the same buffer for 3 h at 4°C using a dialysis membrane (Spectra/Por 7: molecular cut-off 50,000; Spectrum) and then against a new batch of the same buffer overnight. The dialyzed sample was loaded onto Q Sepharose resin (1 ml) equilibrated with the same buffer. After being washed with the same buffer (10 ml), proteins were eluted in a step-wise manner with buffer C including 100, 250, 500, or 1000 mM KCl (5 ml, each). Eluates with 250 and 500 mM KCl were combined and diluted by four times with buffer C and then applied onto a P11 column (1 ml) equilibrated with buffer C containing 100 mM KCl. The resin was washed with the same buffer (10 ml), and further washed with the same buffer

without glycerol (10 ml). The proteins were eluted out with a buffer (5 ml; 20 mM HEPES-KOH pH 7.5, 600 mM KCl, 1 mM DTT, 0.1 mM EDTA). Eluates were concentrated with Amicon Ultra-15 (molecular cut-off 50,000) to approximately 1 ml, and then resolved by 10-30% (w/v) sucrose-gradient centrifugation with a buffer (20 mM HEPES-KOH pH 7.5, 500 mM KCl, 1 mM DTT, 0.1 mM EDTA) at 38,000 rpm in the SW41 rotor for 24 h at 4°C. Five hundred-microliter fractions were successively taken from the top of the gradient, and each fraction was monitored by SDS-PAGE followed by CBB staining and by western blot with antisera against p28, p47, and p110 [8] for the presence of eIF3. The main fractions that contained eIF3 were combined, and were concentrated using Amicon Ultra-15 (molecular cut-off 50,000) to approximately 1 ml. This sample was again resolved by the 10-30% (w/v) sucrose-gradient centrifugation in the same conditions as described above. The fractions that contained eIF3 were dialyzed against 100-times volume of a buffer (20 mM HEPES-KOH pH 7.5, 100 mM KCl, 10% glycerol, 1 mM DTT) for 3 h at 4°C, and then against a new batch of the same buffer overnight. The sample was finally concentrated as stated above.

Recombinant eIF4G/eIF4E complex: BHK-21 cells (thirty confluent 150-mm dishes) infected with the vaccinia virus (LO-T7-1) [9] were transfected with pUC-T7-EMCV-FLAG-eIF4G (84-1599)-His or pUC-T7-EMCV-FLAG-eIF4G (197-1599)-His [10]. Two days later, the cells were harvested and lysed in buffer D [15 ml; 20 mM HEPES-KOH pH 7.5, 500 mM KCl, 10% glycerol, 0.5% Triton X-100, 5 mM β-mercaptoethanol, 1 mM EDTA, 1×protease inhibitor cocktail EDTA-free (Nacalai)]. After centrifugation at 15,000 ×*g* for 30 min at 4°C, the supernatant was supplemented with imidazole to 20 mM and then loaded onto a Ni-NTA resin (1 ml). The resin was washed with a buffer (10 ml; 20 mM HEPES-KOH pH 7.5, 1 M KCl, 10% glycerol, 0.1% Triton X-100, 5 mM β-mercaptoethanol, 1 mM EDTA, 20 mM imidazole) and further washed with the same buffer (10 ml) that reduced the concentration of KCL to 100 mM. Bound proteins were eluted in a stepwise manner with increasing concentrations of imidazole (50, 100, 250, and 1000 mM) in a buffer (20 mM HEPES-KOH pH 7.5, 100 mM KCl, 10% glycerol, 0.1% Triton X-100, 5 mM β-mercaptoethanol, 1 mM EDTA) (3 ml, each). The eIF4G-containing fractions determined by SDS-PAGE followed by CBB-staining were combined and applied onto SP Sepharose chromatography (1 ml, GE Healthcare). After being washed with a buffer (10 ml; 20 mM HEPES-KOH pH 7.5, 100 mM KCl, 10% glycerol, 2.5 mM β-mercaptoethanol, 0.1 mM EDTA), the proteins were eluted with a buffer (5 ml; 20 mM HEPES-KOH pH 7.5, 1 M KCl, 10% glycerol, 2.5 mM β-mercaptoethanol, 0.1 mM EDTA). The eluates were concentrated by Amicon Ultra-15 (molecular cut-off 50,000) to approximately 2.5 ml and then loaded onto a PD-10 column

equilibrated with 20 mM HEPES-KOH pH 7.5, 100 mM KCl, 10% glycerol. After elution with the same buffer (3.5 ml), the eluate was mixed with non-tagged eIF4E (approximately 0.5 mg) and incubated at room temperature for 30 min. The mixture was applied onto anti-FLAG M2 resin (0.2 ml). The resin was washed with the same buffer (4 ml). Bound proteins were incubated with the 1×FLAG peptide (150 ng/μl) in the same buffer (0.6 ml) for 10 min and drained out from the column. The eluates were concentrated with Amicon Ultra-15 (molecular cut-off 50,000).

Recombinant eIF5B: BHK-21 cells (thirty confluent 150-mm dishes) infected with the vaccinia virus (LO-T7-1) [7] were transfected with pUC-T7-EMCV-His-eIF5B-FLAG. Two days later, the cells were harvested and lysed in buffer E (15 ml; 20 mM HEPES-KOH pH 7.5, 100 mM KCl, 10% glycerol, 0.1% Triton X-100, 2 mM β-mercaptoethanol, 1 mM EDTA, 10 mM imidazole, 1×protease inhibitor cocktail EDTA-free). After centrifugation at 15,000 ×*g* for 30 min at 4°C, the supernatant was loaded onto a Ni-NTA resin (1 ml). After being washed with a buffer (10 ml; 20 mM HEPES-KOH pH 7.5, 0.5 M KCl, 10% glycerol, 2 mM β-mercaptoethanol, 1 mM EDTA, 10 mM imidazole) and further washed with the same buffer (10 ml) that reduced the concentration of KCl to 100 mM. The proteins were eluted in a stepwise manner with increasing concentrations of imidazole (50, 100, 250, and 1000 mM) in a buffer (20 mM HEPES-KOH pH 7.5, 100 mM KCl, 10% glycerol, 2 mM β-mercaptoethanol, 1 mM EDTA) (3ml, each). The eIF5B-containing fractions determined by CBB-stained SDS-PAGE were applied onto SP Sepharose resin (1 ml). After the resin was washed with a buffer (10 ml; 20 mM HEPES-KOH pH 7.5, 100 mM KCl, 10% glycerol, 2 mM β-mercaptoethanol, 1 mM EDTA), the proteins were eluted with a buffer (5 ml; 20 mM HEPES-KOH pH 7.5, 500 mM KCl, 10% glycerol, 2 mM β-mercaptoethanol, 1 mM EDTA). The eluate was concentrated by Amicon Ultra-15 (molecular cut-off 50,000) to approximately 2.5 ml and then loaded onto a PD-10 column equilibrated with 20 mM HEPES-KOH pH 7.5, 100 mM KCl, 10% glycerol. After elution with the same buffer (3.5 ml), the eluate was applied onto anti-FLAG M2 resin (0.2 ml) and eIF5B was purified as described in "Recombinant eIF4G/eIF4E complex" section. The final product was concentrated with Amicon Ultra-15 (molecular cut-off 50,000).

Recombinant DHX29: BHK-21 cells (confluent on thirty 150-mm dishes) infected with the vaccinia virus (LO-T7-1) [7] were transfected with pUC-T7-EMCV-His-DHX29. After two days, the cells were harvested and lysed in buffer E (15 ml). After centrifugation at 15,000 ×*g* for 30 min at 4°C, the supernatant was subjected to a Ni-NTA chromatography (1 ml) as described above (see the "Recombinant eIF4G/eF4E complex" section). The DHX29-containing fractions determined by CBB-stained SDS-PAGE were

applied onto SP Sepharose resin (0.4 ml). The proteins were eluted out in a step-wise manner that increased the concentration of KCl from 100 to 1000 mM in a buffer (1.2 ml each; 20 mM HEPES-KOH pH 7.5, 500 mM KCl, 10% glycerol, 2 mM β-mercaptoethanol, 1 mM EDTA). The eluates with 200, 300, 400, and 500 mM KCl were combined and concentrated by Amicon Ultra-15 (molecular cut-off 50,000) to approximately 2.5 ml and then loaded onto a PD-10 column equilibrated with 20 mM HEPES-KOH pH 7.5, 100 mM KCl, 10% glycerol. After elution with 3.5 ml of the same buffer, the eluate was concentrated as the DHX29 protein preparation.

Recombinant ABCE1: ABCE1-3XFLAG was expressed and purified as described [11].

Recombinant eEF1: Purification of His-eEF1 was performed as described [12] with following modifications: frozen BHK-21 cells from sixty confluent 150-mm dishes expressing His-eEF1A, eEF1Bα, and His-eEF1Bγ were lysed and centrifuged. The supernatant was mixed with NaCl to the final concentration of 1 M and then passed through SP Sepharose resin (3 ml) to remove possibly contaminating polyethyleneimine, the reagent used for transfection. The unbound fraction was subjected to Ni-NTA chromatography (3 ml). The eEF1-containing fractions were combined, mixed with NaCl, and applied onto SP seharose (2 ml) as described above. The unbound fraction was concentrated using Amicon Ultra-15 (molecular weight cut-off 50,000) to approximately 2.5 ml and then loaded onto a PD-10 column equilibrated with a buffer (20 mM HEPES-KOH pH 7.5, 100 mM KCl, and 10% glycerol). After elution with 3.5 ml of the same buffer, the eEF1 preparation was concentrated as stated above.

Recombinant aminoacyl-tRNA synthetases (ARSs): The LO-T7-1-infected BHK-21 cells (thirty confluent 150-mm dishes) were co-transfected with the following 23 plasmids (47 μg each): pUC-T7-EMCV-His-Glu-ProRS, -His-IleRS, -His-LeuRS, -His-MetRS, -His-GlnRS, -His-LysRS, -His-ArgRS, -His-AspRS, -His-AlaRS, -His-CysRS, -His-GlyRS, -His-HisRS, -His-AsnRS, -His-SerRS, -His-ThrRS, -His-ValRS, -His-TrpRS, -His-TyrRS, -His-PheRSα, and -His-PheRSβ and pUC-T7-EMCV p18-His, -p38-His, and -p43-His [12]. The transfected BHK-21 cells were lysed and applied onto Ni-NTA chromatography (1 ml) as described in “Recombinant eIF2B” section. The eluates with 50, 100, 250 mM imidazole were combined and concentrated using Amicon Ultra-15 (molecular weight cut-off 50,000) to approximately 2.5 ml and then loaded onto a PD-10 column equilibrated with a buffer (20 mM HEPES-KOH pH 7.5, 100 mM KCl, and 10% glycerol). After elution with 3.5 ml of the same buffer, the ARSs sample was concentrated as stated above.

The protein concentration of each sample was determined by Bradford assay.

Antibodies: Anti-eIF4GI was obtained from a rabbit immunized with GST-eIF4GI (172-

200). Anti-S6 (Cell Signaling), L13a (Cell Signaling) and PABP (Abcum ab21060: an antibody to a C-terminal region of human PABP) were purchased.

RNA

Purification of tRNAs: Total tRNAs were obtained from HeLa cells as previously described [12].

For the synthesis of a poly(A)-tailed mRNA, the template plasmid (pUC-T7-HA-Rluc-myc-polyA$_{118}$) was linearized by digestion with *Xho*I and purified with phenol/chloroform extraction followed by ethanol precipitation. For the preparation of a poly(A)-minus version of the mRNA, the same plasmid was digested with *Xba*I and purified as described above. To synthesize a capped mRNA, the linearized template (12 μg) was mixed with a reaction buffer [50 μl, total volume; 80 mM HEPES-KOH pH 7.5, 24 mM magnesium chloride, 2 mM spermidine trihydrochloride, 40 mM DTT, 5 mM ATP, 5 mM CTP, 5 mM UTP, 0.8 U/μl SUPERase In RNase inhibitor (ThermoFisher), 2 U/μl T7 RNA polymerase (Promega)] with 1 mM GTP and 4 mM m^{7}G(5')ppp(5')G RNA Cap Structure analog (New England Biolabs). The sequence of the 5' untranslated region of this RNA was m^{7}G(5')ppp(5')GGAUCCACCGCC. To synthesize an uncapped mRNA, the linearized DNA (12 μg) was mixed with the same reaction buffer but with 5 mM GTP in place of 1 mM GTP and 4 mM m^{7}G(5')ppp(5')G. Each mixture was incubated at 37°C for 3 h and then treated with TURBO DNase (4 U: ThermoFisher) at 37°C for 30 min. After incubation, the reaction sample was mixed with 5 M ammonium acetate/0.1 M EDTA (30 μl) and RNase-free water (216 μl) and purified with phenol/chloroform extraction followed by isopropanol precipitation. The RNA pellet was dissolved in the RNA storage solution (30 μl, ThermoFisher:) and further purified using Chroma Spin +TE-30 (Clontech). The concentration of each RNA was determined by measuring OD at 260 nm. Their integrity was examined by electrophoresis on formaldehyde-agarose gels.

In vitro dissociation experiment

To carry out the dissociation experiment (Figure 2D), a slurry (approximately 10 μl) of the TALON resin (TaKaRa) equilibrated with an equilibrium buffer (20 mM Tris-HCl pH7.5, 1 M KCl, 4 mM magnesium acetate, 10% glycerol, 0.1 % Triton-X100) was mixed with the same buffer (30 μl) containing bovine serum albumin (BSA, 1 mg/ml), and incubated for 30 min at 4°C. PABP-His (15 pmol) was added to the resin, and the incubation was continued for 30 min at 4°C. The resin was then centrifuged at 5000 g for 1 min at 4°C and the supernatant was removed. The resin was washed with the equilibrium buffer (200 μl) five times to prepare a PABP-His-resin.

The PABP-His-resin was incubated with 40S or 60S subunit (7.5 pmol, each) in a ribosome binding buffer (30 µl; 20 mM Tris-HCl pH7.5, 0.1 M KCl, 4 mM magnesium acetate, 10% glycerol, 0.1 % triton-X100) for 30 min at 4°C. The resin was washed with the ribosome binding buffer (200 µl) five times to prepare a 40S or 60S ribosomal subunit-PABP-His resin.

The 40S or 60S subunit-PABP-His resin was incubated with the poly(A) RNA or HA-Rluc-N RNA (0, 3 or 30 pmol) in the ribosome binding buffer (30 µl) for 5 min at 4°C. The resin was centrifuged at 5000 g for 1 min at 4°C and the supernatant (sample 1) was harvested. The resin was then washed with the ribosome binding buffer (200 µl), and RNA/proteins that were still retained on the resin (sample 2) were eluted with the ribosome binding buffer (30 µl) containing 500 mM imidazole. Aliquots of sample 1 and 2 (5 µl, each) were analyzed by SDS-PAGE followed by western blotting with anti-PABP, anti-S6 and anti-L13a antibodies.

CRAC analysis

To determine the PABP binding sites on the ribosome, cross-linking and analysis of cDNA (CRAC) [13,14] was performed. PABP-His-PA (60 pmol) was incubated with the 40S or 60S ribosomal subunit (60 pmol, each) in a ribosome binding buffer (50 µl; 20 mM Tris-HCl pH7.5, 0.1 M KCl, 4 mM magnesium acetate, 10% glycerol, 0.1 % triton-X100) for 30 min at 4°C. Each sample was then irradiated with 1.92 J/cm^2 of ultraviolet (254 nm) in a CL1000 Ultraviolet Crosslinker (Biostad).

A slurry (approximately 20 µl) of the Ni-NTA agarose resin (QIAGEN) equilibrated with a CRAC washing buffer (500 µl; 50 mM Tris-HCl pH6.8, 10 mM imidazole, 300 mM NaCl, 0.1% NP-40, 5 mM β-mercaptoethanol, 6 M guanidine hydrochloride) was mixed with the irradiated sample (50 µl) in the same buffer, and incubated for 3 h at 4°C for denaturation and binding of the PABP-His-PA-containing complexes to the resin. Then, the resin was centrifuged at 5000 g for 1 min at 4°C, and the supernatant was removed.

The resin was then washed with a PNK buffer (500 µl; 50 mM Tris-HCl pH 7.6, 10 mM $MgCl_2$, 0.5% NP-40, 5 mM β-mercaptoethanol) three times, and incubated with the same buffer (20 µl) containing RNace-IT (1.25 U RNase T1, 0.5 µg RNase A; Agilent) for 5 min at 37°C. The CRAC washing buffer (500 µl) was added to the sample, and centrifuged at 5000 g for 1 min at 4°C. After removal of the supernatant, the resin was washed with the PNK buffer (500 µl), and incubated with an elution buffer (20 µl; 50 mM Tris-HCl pH7.6, 50 mM NaCl, 200 mM imidazole, 0.1% NP-40, 5 mM β-mercaptoethanol) for 20 min at 4°C and centrifuged at 5000 g for 1 min at 4°C.

An aliquot of the supernatant (2 µl) was analyzed by 4-12% Bis-Tris Gel NuPAGE (Invitrogen) followed by western blotting with the anti-PA antibody to determine the size of the UV-cross linked products. The remaining sample was separated by 4-12% Bis-Tris Gel NuPAGE and transferred to a nitrocellulose membrane. From the membrane area that were considered to contain the complexes with PABP-His-PA, RNA was extracted by incubation with a buffer (400 µl; 50 mM Tris-HCl pH7.6, 50 mM NaCl, 1% SDS, 5 mM EDTA, 0.1% NP-40, 5 mM β-mercaptoethanol, 4 µg Proteinase K) for 2 h at 55°C. After incubation, RNA was purified with phenol/chloroform extraction followed by ethanol precipitation.

The purified RNA dissolved in the RNA storage solution (72 µl; Ambion) was mixed with MULTI-CORE buffer (8.8 µl, Promega) containing TSAP (8 U, Promega), and incubated with for 15 min at 37 °C. The sample was purified with phenol/chloroform extraction followed by ethanol precipitation, and the RNA pellet was dissolved in the RNA storage solution (7 µl).

Polyadenylation, cDNA synthesis and the 1st PCR for amplification of cDNAs were performed using SMARTer smRNA-Seq Kit for Illumina (TaKaRa). The 1st PCR products were separated on 2% Agarose-LM (Nacalai Tesque) and stained with ethidium bromide, and the PCR products with the insert size between 200 and 500 base pairs (bp) were purified. Then the 2nd PCR was performed to amplify the purified 1st PCR products using the same primer as for the 1st PCR but with the Ex-Taq (TaKaRa) enzyme. The PCR products with the insert size between 200 and 500 bp were purified.

The purified 2nd PCR products were cloned in the T-Vector pMD20 (TaKaRa) for sequencing.

Supplementary References

1 Imataka, H., Gradi, A. & Sonenberg, N. A newly identified N-terminal amino acid sequence of human eIF4G binds poly(A)-binding protein and functions in poly(A)-dependent translation. *The EMBO journal* 17, 7480-7489 (1998).

2 Khaleghpour, K. *et al.* Dual interactions of the translational repressor Paip2 with poly(A) binding protein. *Mol Cell Biol* 21, 5200-5213, doi:10.1128/MCB.21.15.5200-5213.2001 (2001).

3 Mikami, S., Masutani, M., Sonenberg, N., Yokoyama, S. & Imataka, H. An efficient mammalian cell-free translation system supplemented with translation factors. *Protein Expr Purif* 46, 348-357 (2006b).

4 Mikami, S., Masutani, M., Sonenberg, N., Yokoyama, S. & Imataka, H. An efficient

mammalian cell-free translation system supplemented with translation factors. *Protein expression and purification* 46, 348-357, doi:10.1016/j.pep.2005.09.021 (2006).

5 Svitkin, Y. V. & Sonenberg, N. An efficient system for cap- and poly(A)-dependent translation in vitro. *Methods Mol Biol* 257, 155-170, doi:10.1385/1-59259-750-5:155 (2004).

6 Machida, K. *et al.* A Translation System Reconstituted with Human Factors Proves That Processing of Encephalomyocarditis Virus Proteins 2A and 2B Occurs in the Elongation Phase of Translation without Eukaryotic Release Factors. *Journal of Biological Chemistry* 289, 31960-31971, doi:10.1074/jbc.M114.593343 (2014).

7 Machida, K. *et al.* Reconstitution of the human chaperonin CCT by co-expression of the eight distinct subunits in mammalian cells. *Protein Expr Purif* 82, 61-69 (2012).

8 Masutani, M., Sonenberg, N., Yokoyama, S. & Imataka, H. Reconstitution reveals the functional core of mammalian eIF3. *The EMBO journal* 26, 3373-3383 (2007).

9 Masutani, M., Machida, K., Kobayashi, T., Yokoyama, S. & Imataka, H. Reconstitution of eukaryotic translation initiation factor 3 by co-expression of the subunits in a human cell-derived in vitro protein synthesis system. *Protein Expr Purif* 87, 5-10, doi:10.1016/j.pep.2012.10.001 (2013).

10 Yanagiya, A. *et al.* Requirement of RNA binding of mammalian eukaryotic translation initiation factor 4GI (eIF4GI) for efficient interaction of eIF4E with the mRNA cap. *Mol Cell Biol* 29, 1661-1669, doi:10.1128/MCB.01187-08 (2009).

11 Machida, K. *et al.* Huntingtin Polyglutamine-Dependent Protein Aggregation in Reconstituted Cells. *ACS Synth Biol* 7, 377-383, doi:10.1021/acssynbio.7b00372 (2018).

12 Machida, K. *et al.* A translation system reconstituted with human factors proves that processing of encephalomyocarditis virus proteins 2A and 2B occurs in the elongation phase of translation without eukaryotic release factors. *J Biol Chem* 289, 31960-31971, doi:10.1074/jbc.M114.593343 (2014).

13 Granneman, S., Kudla, G., Petfalski, E. & Tollervey, D. Identification of protein binding sites on U3 snoRNA and pre-rRNA by UV cross-linking and high-throughput analysis of cDNAs. *Proc Natl Acad Sci U S A* 106, 9613-9618, doi:10.1073/pnas.0901997106 (2009).

14 Granneman, S., Petfalski, E., Swiatkowska, A. & Tollervey, D. Cracking pre-40S ribosomal subunit structure by systematic analyses of RNA-protein cross-linking. *The EMBO journal* 29, 2026-2036, doi:10.1038/emboj.2010.86 (2010).

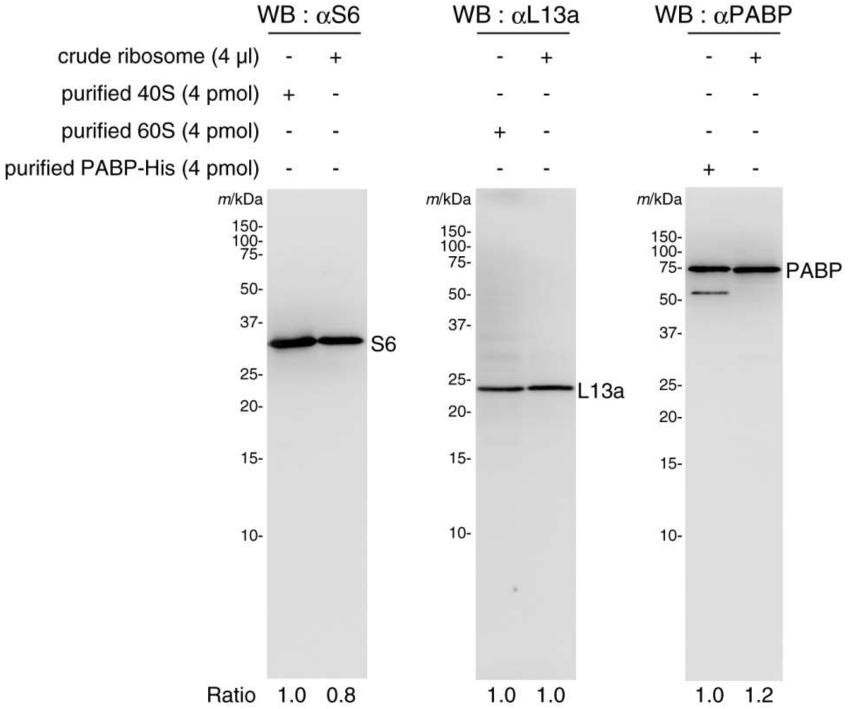

Figure S1. The presence of PABP in the crude ribosomal preparation
The ribosome preparation (crude ribosome) obtained after the first sucrose gradient centrifugation without the gel filtration chromatography was analyzed by western blotting (WB: 40S, 60S and PABP). The highly purified 40S and 60S subunits (4 pmol) and recombinant PABP-His (4 pmol) were loaded as the references.

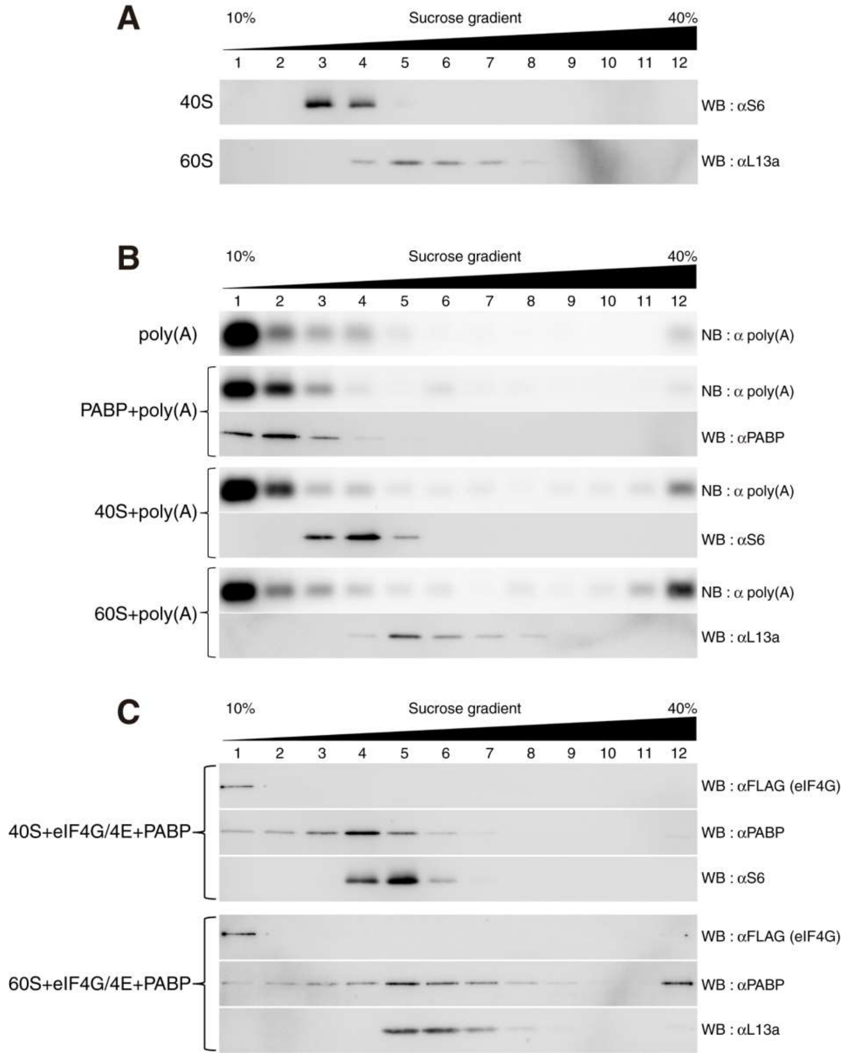

Figure S2. Migration of the ribosomal subunits, the poly(A) RNA and eIF4G/4E on 10–40% sucrose-gradient centrifugation

Fractions after sucrose-gradient centrifugation were analyzed by western blotting (WB: PABP, eIF4G, 40S and 60S) or northern blotting (NB: RNA). (A) The 40S subunit (30 pmol) alone or the 60 S subunit (30 pmol) alone was resolved by sucrose-gradient centrifugation. (B) The poly(A) RNA (60 pmol) alone, PABP (60 pmol) plus the poly(A) RNA (60 pmol), the 40S subunit (30 pmol) plus the poly(A) RNA (60 pmol), or the 60S subunit (30 pmol) plus the poly(A) RNA (60 pmol) was resolved by sucrose-gradient centrifugation. (C) The 40S subunit (30 pmol) plus eIF4G/4E (60 pmol) plus the PABP (60 pmol) or the 60S subunit (30 pmol) plus eIF4G/4E (60 pmol) plus the PABP (60 pmol) was resolved by sucrose-gradient centrifugation. FLAG-eIF4G (84–1599)-His was used as eIF4G.

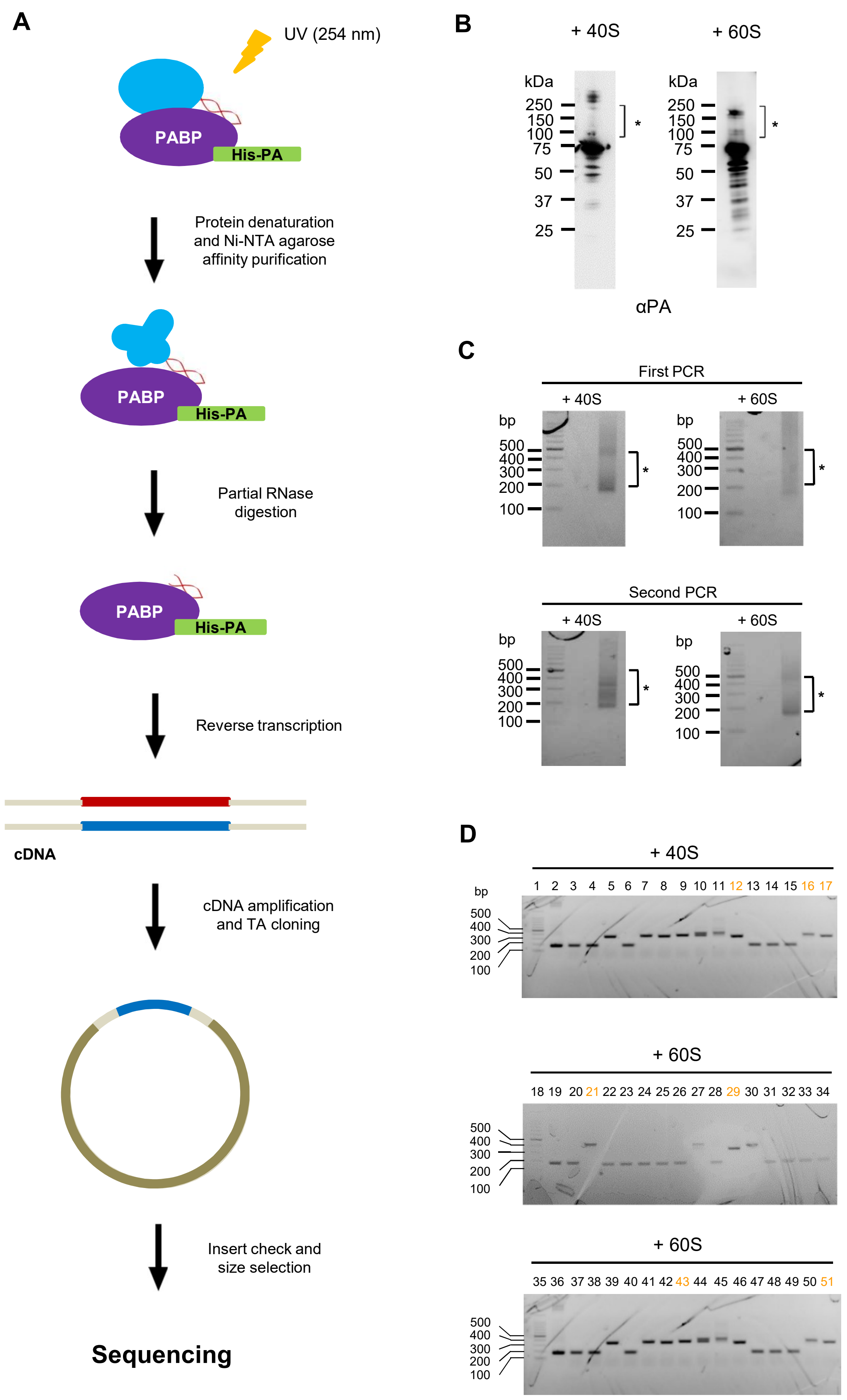

A
UV (254 nm)
PABP
His-PA
Protein denaturation and Ni-NTA agarose affinity purification
PABP
His-PA
Partial RNase digestion
PABP
His-PA
Reverse transcription
cDNA
cDNA amplification and TA cloning
Insert check and size selection
Sequencing
B
+ 40S
+ 60S
kDa
250
150
100
75
50
37
25
*
αPA
C
First PCR
+ 40S
+ 60S
bp
500
400
300
200
100
*
Second PCR
+ 40S
+ 60S
D
+ 40S
1 2 3 4 5 6 7 8 9 10 11 12 13 14 15 16 17
+ 60S
18 19 20 21 22 23 24 25 26 27 28 29 30 31 32 33 34
+ 60S
35 36 37 38 39 40 41 42 43 44 45 46 47 48 49 50 51

Figure S3. CRAC analysis

(A) Schematic diagram of the CRAC method. (B) Western blot analysis of the purified samples containing PABP-His-PA. '*' indicates the membrane area used for RNA extraction. (C) Agarose gel electrophoresis data of the 1st and 2nd PCR. '*' indicates the gel area recovered for the next step. (D) Insert check of the TA-cloned samples. Lane 1, 18 and 35: DNA ladder marker. Lane 2, 19 and 36: no insert. Other lane numbers represent each clone number, and the numbers highlighted in yellow matched the ribosomal RNA sequences (Fig 4). Sequences of other clones were too short to align or did not hit any sequence in the BLAST analysis.

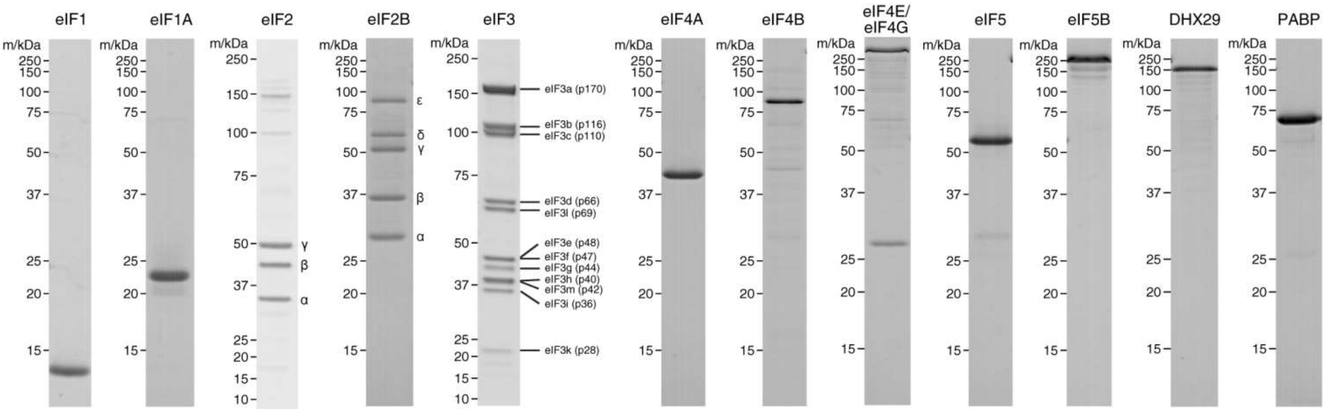

Figure S4. Purified translation initiation factors

eIF1 (1 µg), eIF1A (1 µg), eIF2 (1 µg), eIF2B (1.5 µg), eIF3 (2 µg), eIF4A (1 µg), eIF4B (1 µg), eIF4G (197-1599)/eIF4E (1 µg), eIF5 (1 µg), eIF5B (1 µg), DHX29 (1 µg), and PABP (1 µg) were resolved by SDS-PAGE (13%), and stained with CBB.

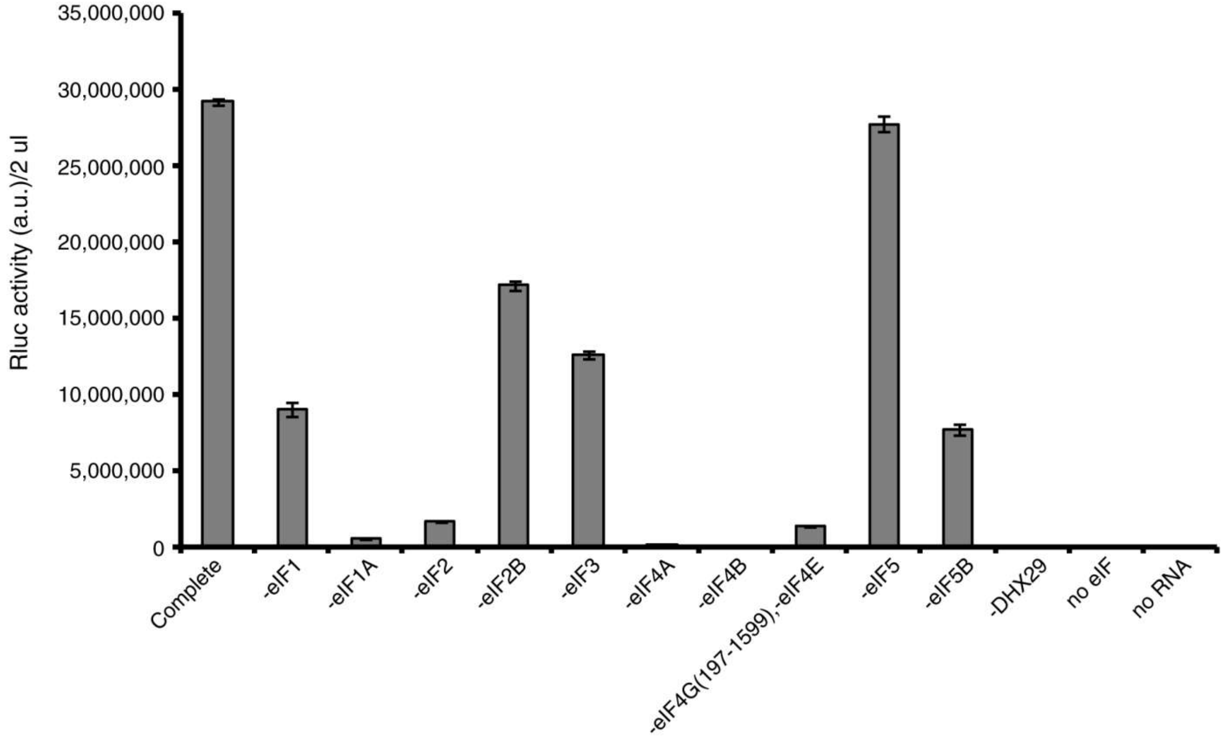

Figure S5. Dependence of the reconstituted translation system on each initiation factor
The translation system was reconstituted with elongation factors, termination factors, ribosomes, tRNAs, aminoacyl tRNA synthetases with all the initiation factors purified in Figure S3 (complete) or with all but the factor indicated. The systems were programmed with Cap-Rluc-A RNA. After incubation, Rluc activity was measured. Each column and bar represent the mean and standard deviation of three experiments, respectively.

Note that PABP was not included .

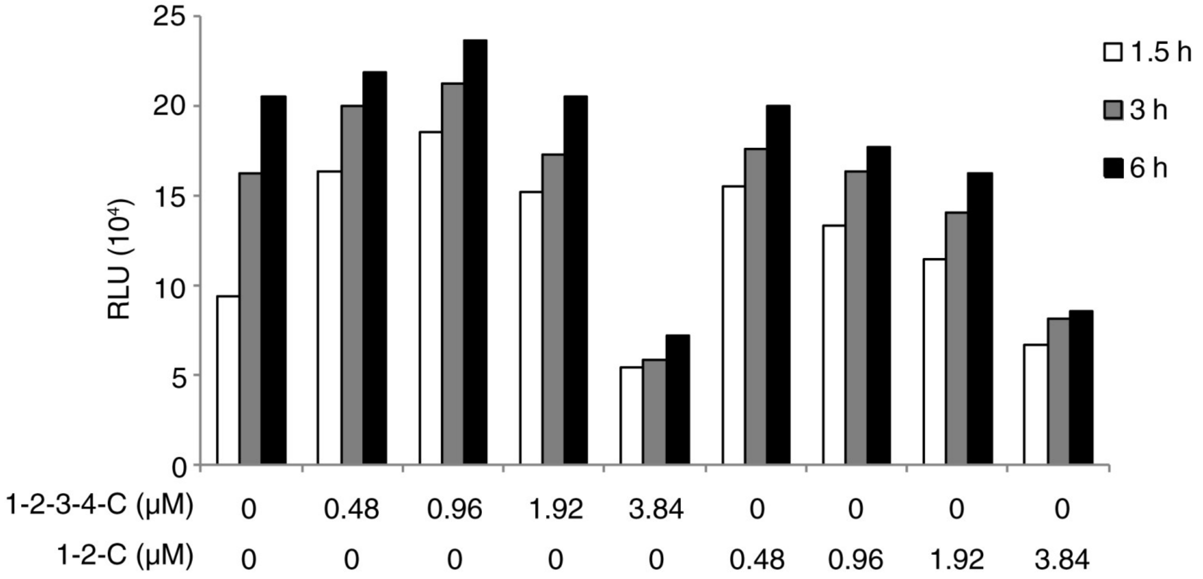

Figure S6. Effects of PABP and a PABP mutant on translation of cap-Rluc RNA
Cap-Rluc RNA (0.1 μM) was translated in the reconstitution system in the presence of increasing concentrations (0 to 3.84 μM) of PABP (1-2-3-4-C) or a truncated PABP (1-2-C). At indicated times, an aliquot of each sample was removed for the Rluc assay. Each bar represents the mean of two experiments.

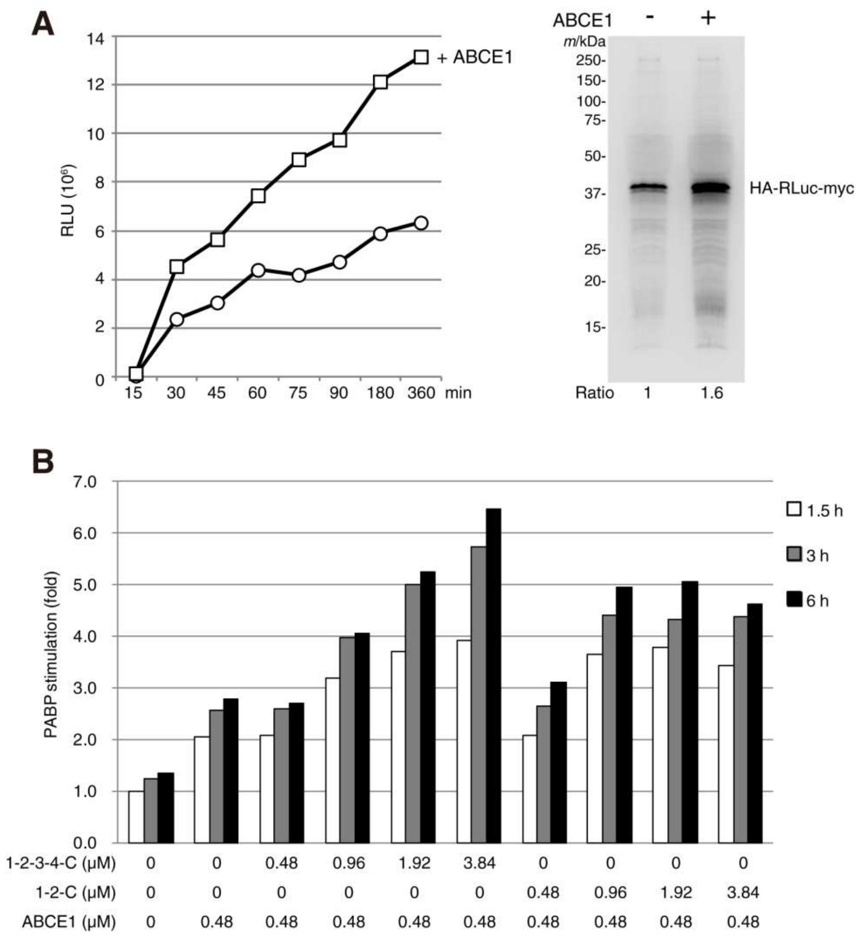

Figure S7. Binding of PABP to the ribosome supports ABCE1-stimulated translation.

(A) The effect of ABCE1 on translation. Cap-Rluc-A RNA (0.1 mM) was translated in the reconstitution system with or without recombinant ABCE1 (0.48 mM); PABP was not included. At the indicated times, an aliquot of each sample was removed for the Rluc assay (left panel). After translation for 6 h, the remaining samples were analyzed by western blotting with an anti-HA antibody (right panel; each HA-Rluc-myc protein band was quantified using ImageJ 1.48v software (https://imagej.nih.gov/ij/), and the relative values were reported below each lane. (B) Cap-Rluc-A RNA (0.1 mM) was translated in the reconstitution system with or without ABCE1 (0.48 mM) in the presence of increasing concentrations (0 to 3.84 mM) of PABP ((1-2-3-4-C) or a PABP mutant (1-2-C). At the indicated times, an aliquot of each sample was removed for the Rluc assay. Each bar represents the mean of two experiments.

Table S1. RNA sequences of RNA fragments used in Fig 2 and 3.

Poly(A):
GGAUCCCAUGGCAUAAUAGGUGUUAAAAAAAAAAAAAAAAAAAAAAAAAAAAAA
AAAAAAAAAAAAAAAAAAAAAAAAAAAAAAAAAAAAAAAAAAAAAAAAAAAAAAAAAAAA
AAAAAAAAAAAAAAAAAAAAAAAAAAAACUCGA (147b)

Note: the “poly(A) RNA” contains non-poly(A) sequences derived from the cloning vector at 5’ and 3’ ends of the 118 A stretch.

HA-Rluc-N:
GGAUCCACCGCCAUGGAAUUUGAAUACCCAUACGAUGUUCCUGACUAUGCGGGCGA
AUUCACUUCGAAAGUUUAUGAUCCAGAACAAAGGAAACGGAUGAUAACUGGUCCGC
AGUGGUGGGCCAGAUGUAAACAAAUGAAUGUUCUCGA (149b)

Unprocessed images

Figure 1, B

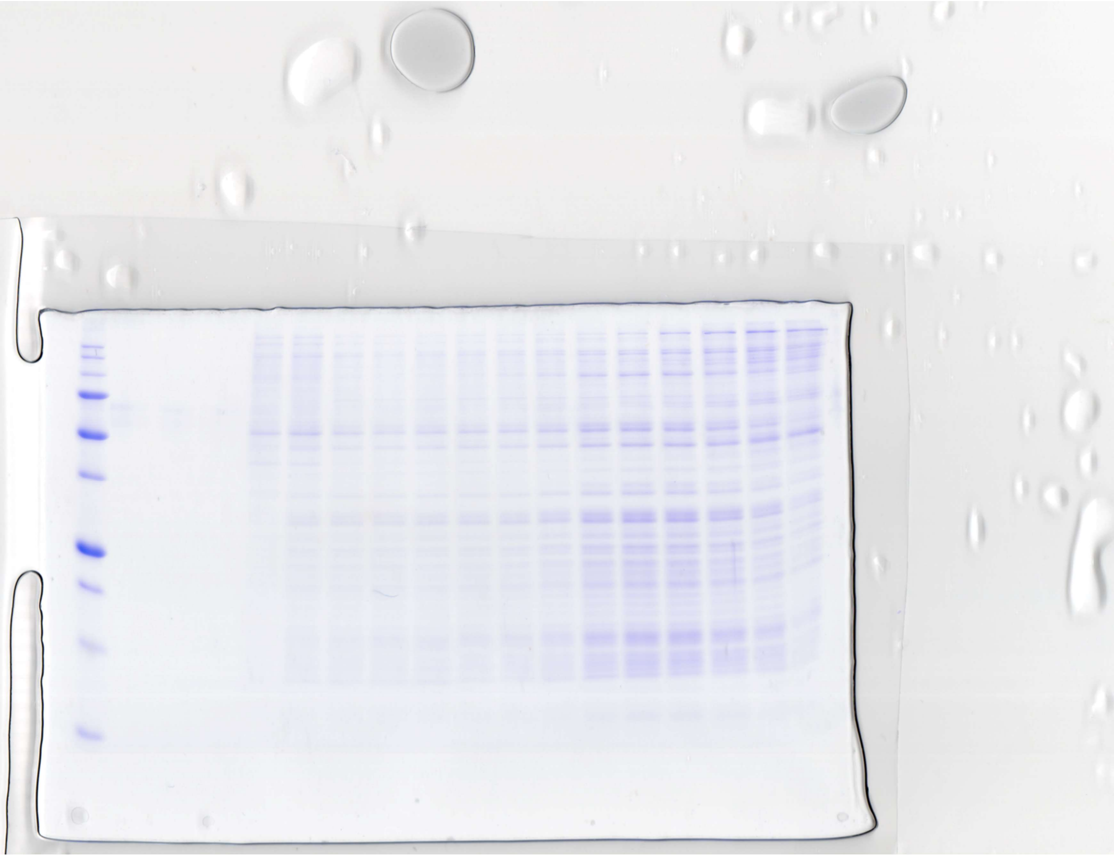

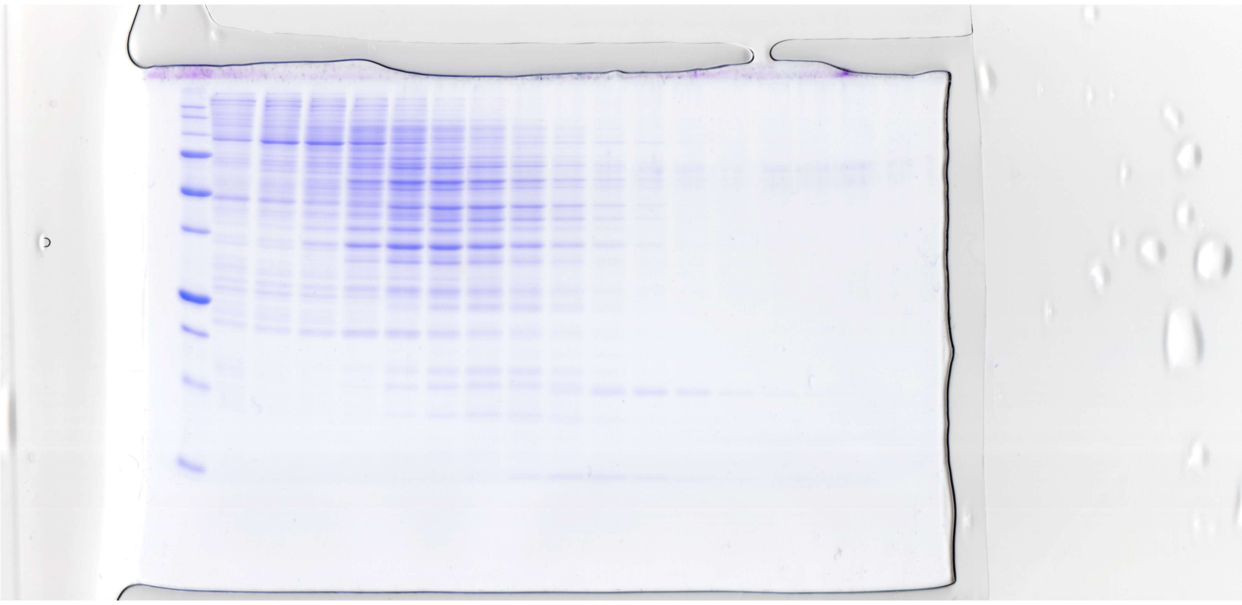

Unprocessed images

Figure 1, B

WB : αS6/αL13a

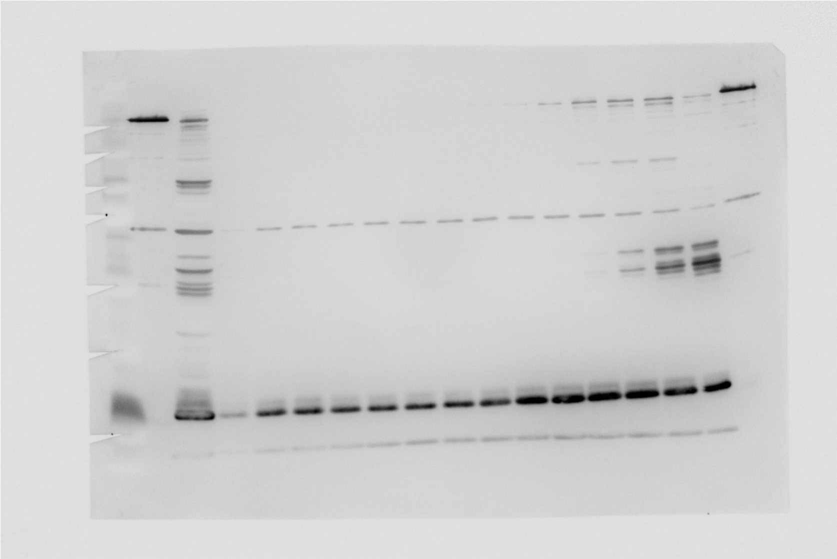

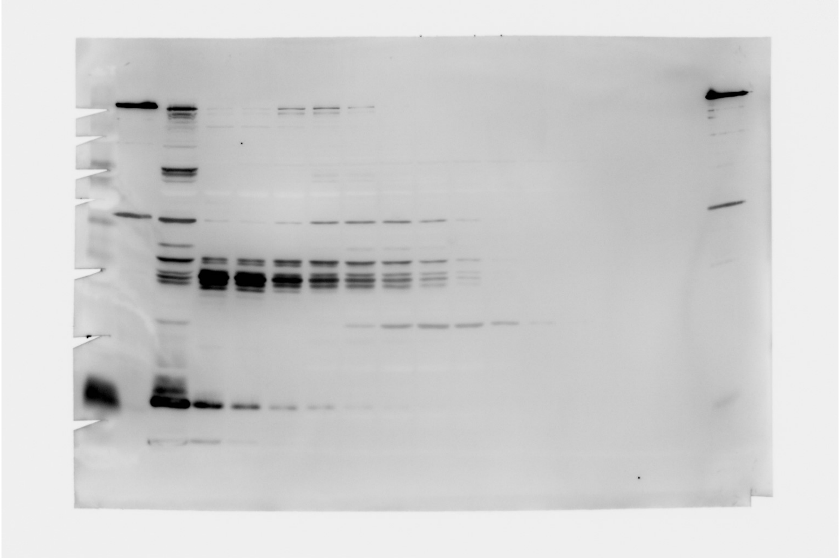

Unprocessed images

Figure 1, B

WB : αPABP

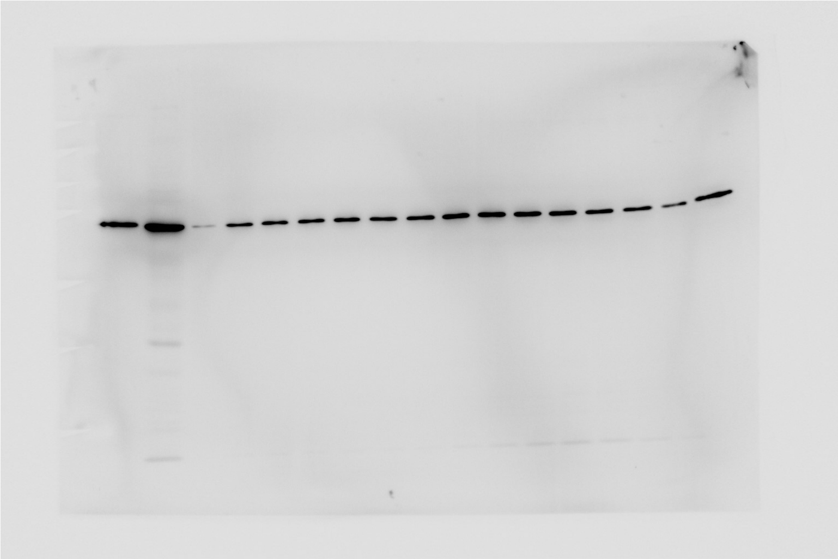

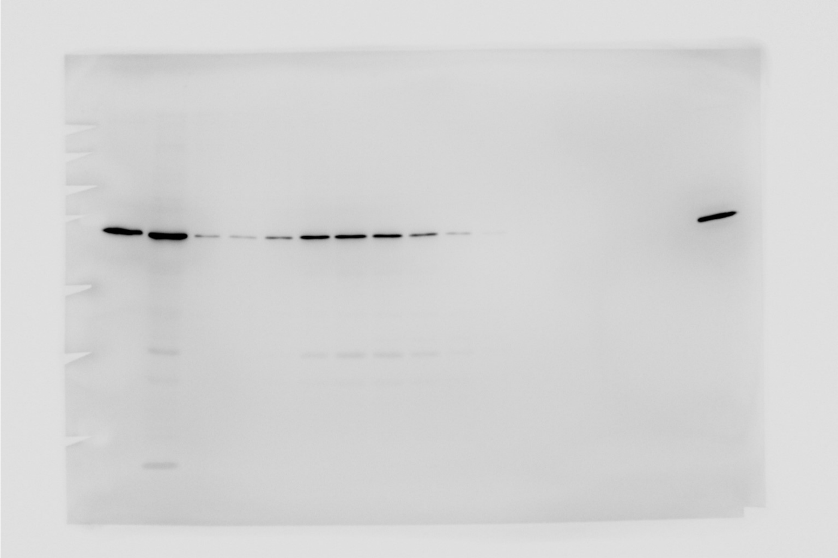

Unprocessed images

Figure 1, B

WB : αeIF4G

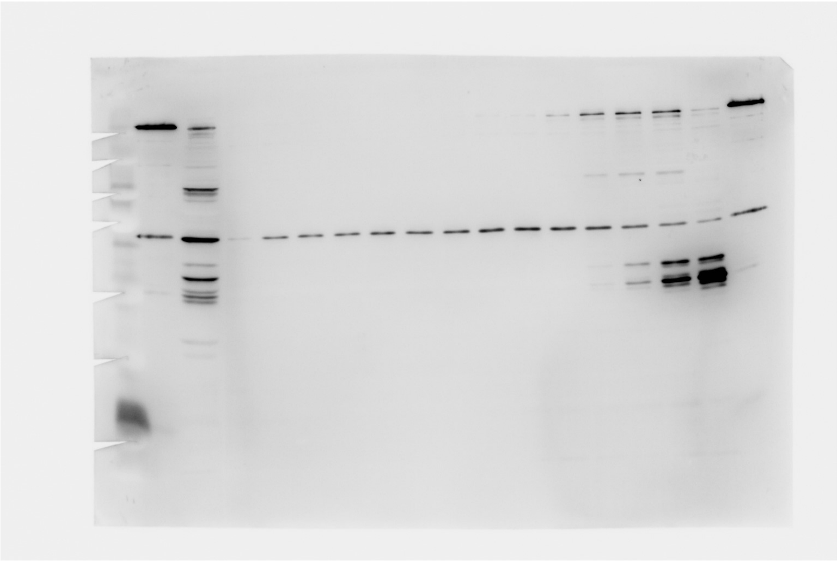

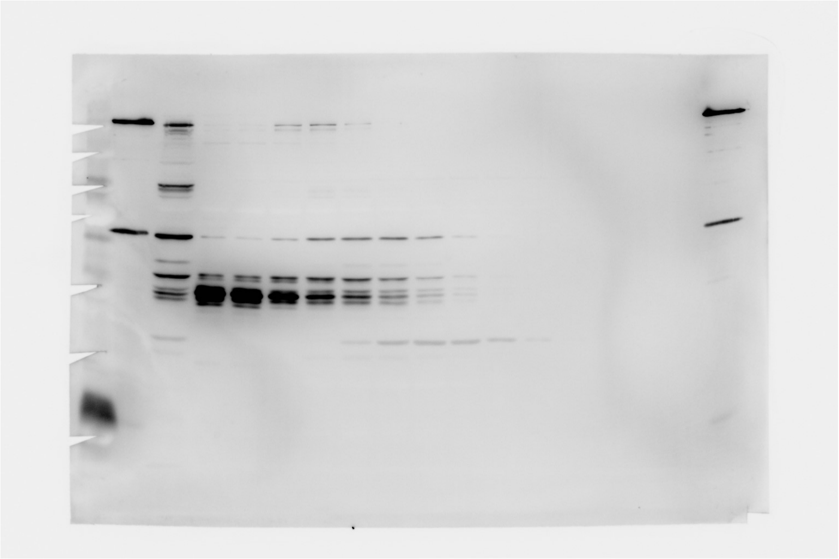

Unprocessed images

Figure 1, C

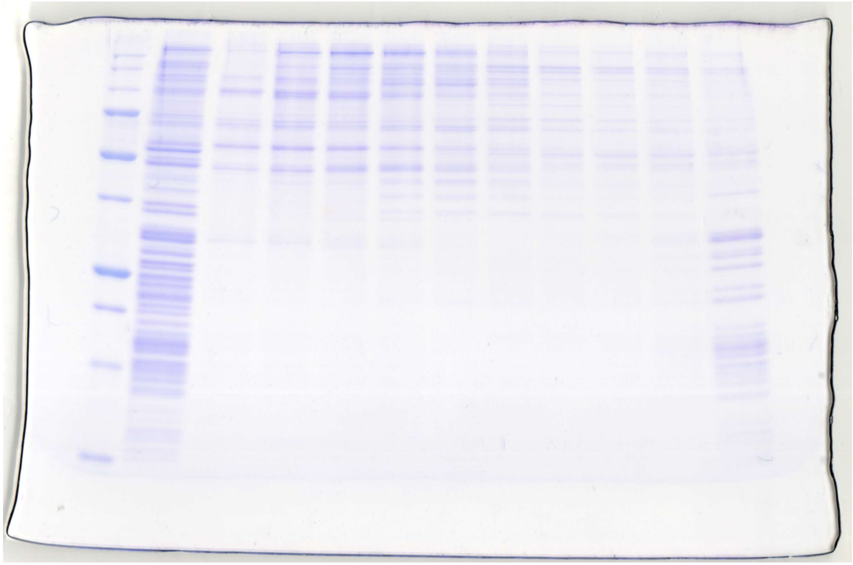

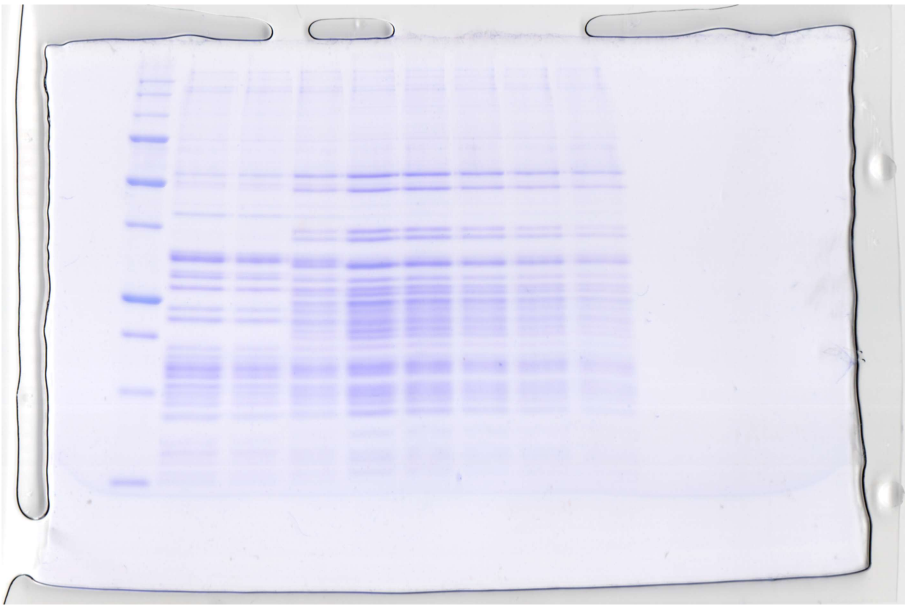

Unprocessed images

Figure 1, C

WB : αS6/αL13a

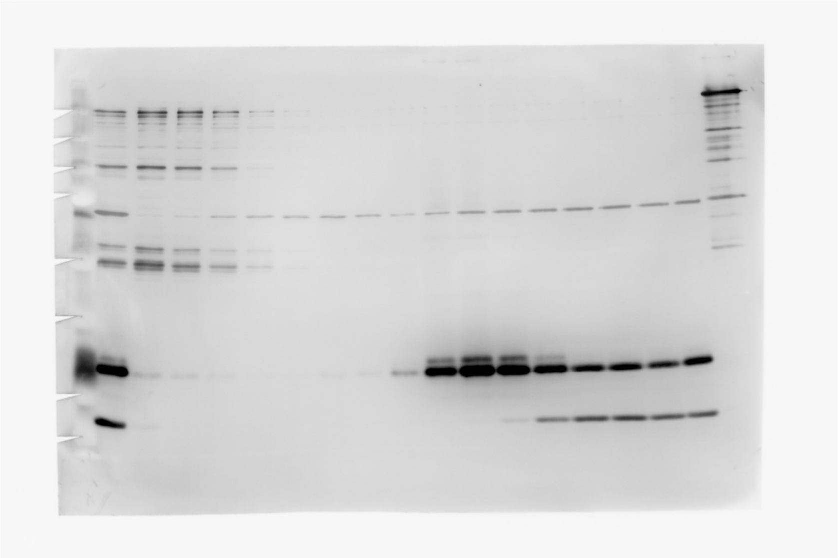

Unprocessed images

Figure 1, C

WB : αPABP

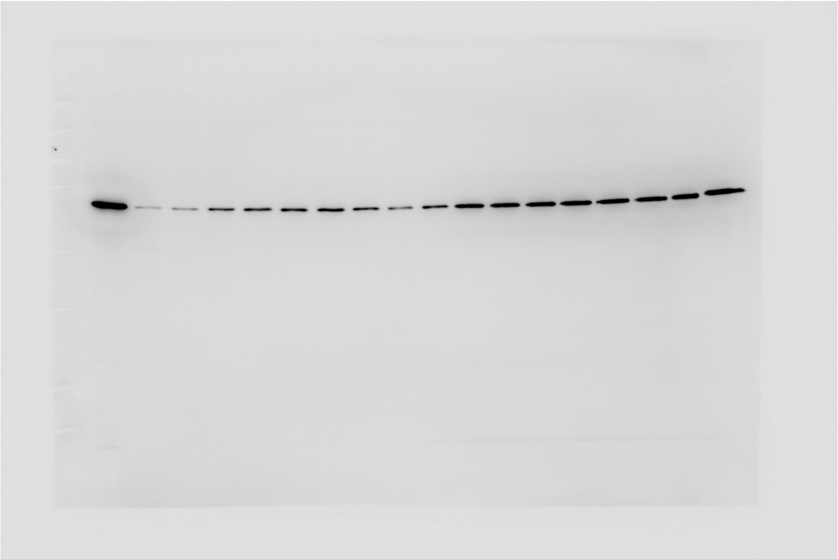

WB : αeIF4G

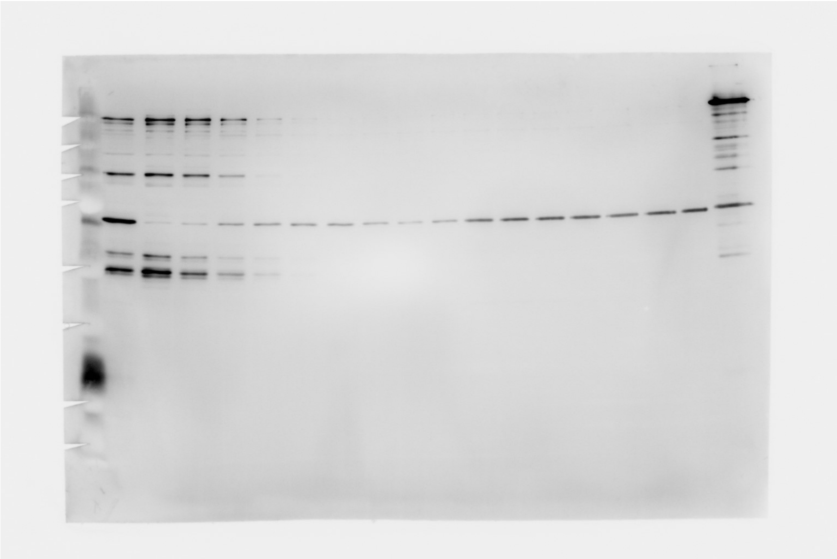

Unprocessed images

Figure 1, D

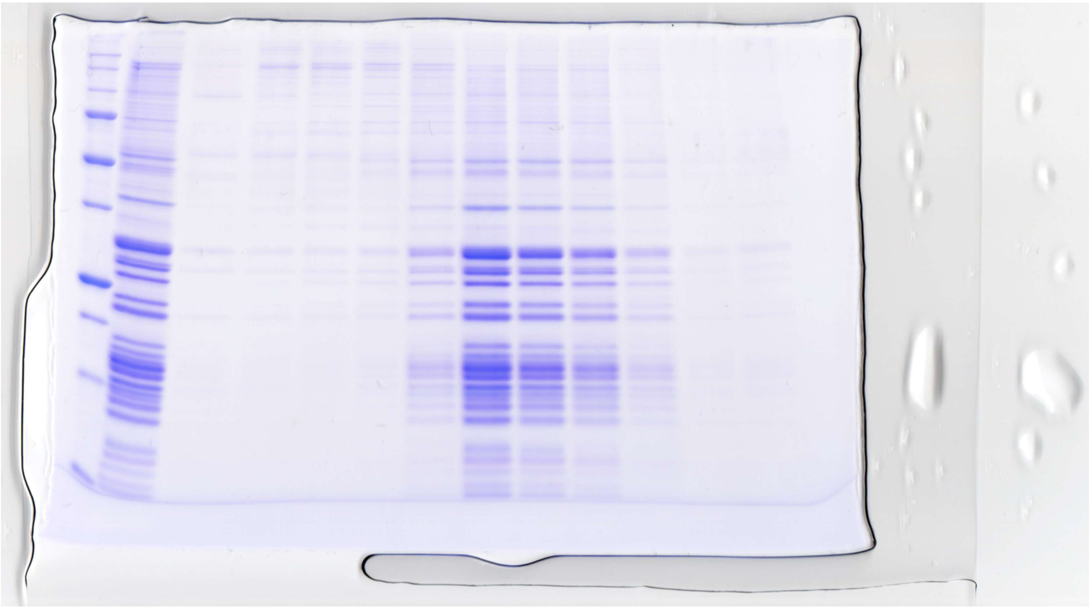

Unprocessed images

Figure 1, D

WB : αS6

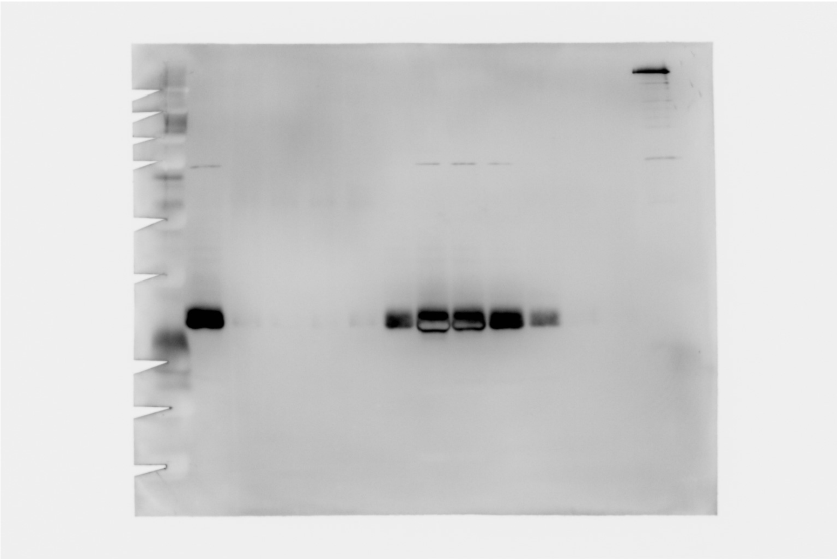

WB : αL13a

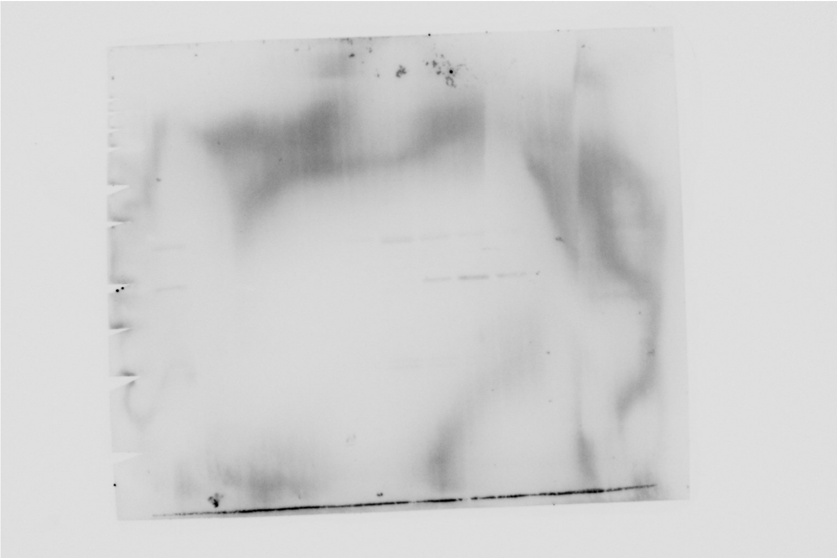

Unprocessed images

Figure 1, D

WB : αPABP

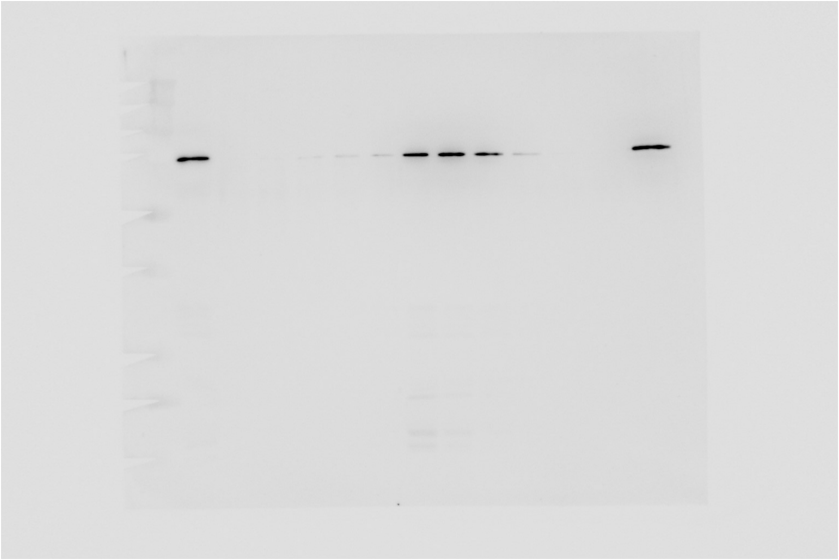

WB : αeIF4G

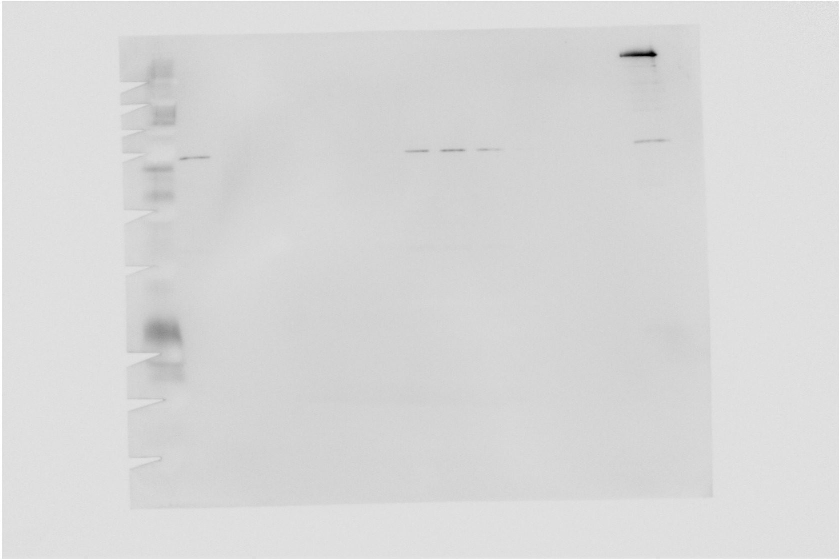

Unprocessed images

Figure 1, E

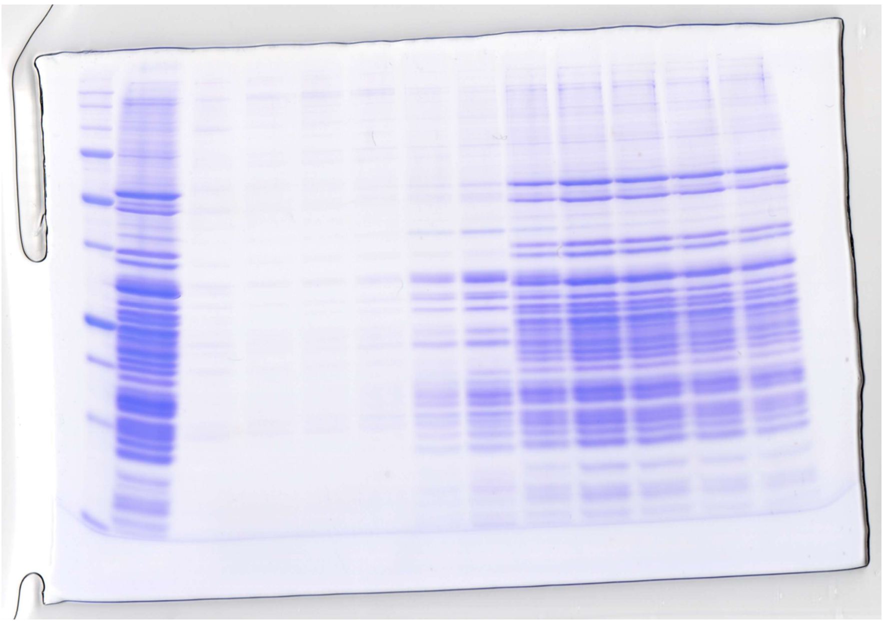

Unprocessed images

Figure 1, E

WB : αS6

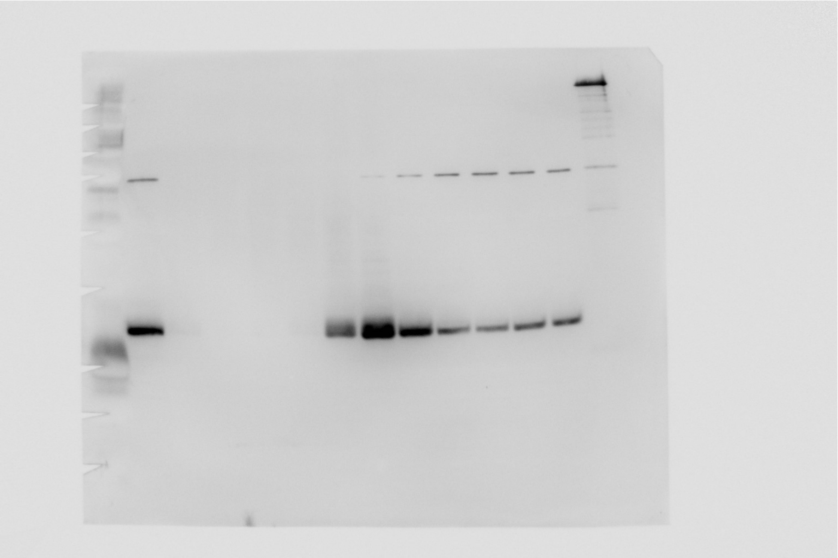

WB : αL13a

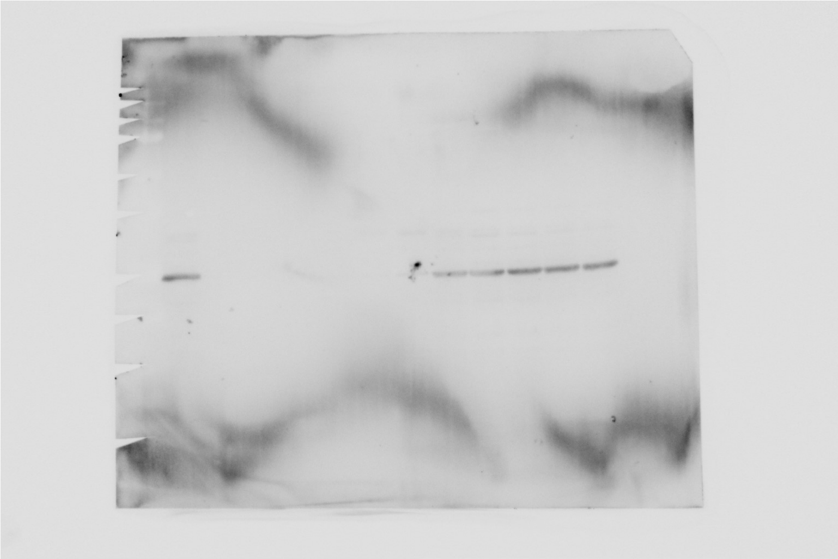

Unprocessed images

Figure 1, E

WB : αPABP

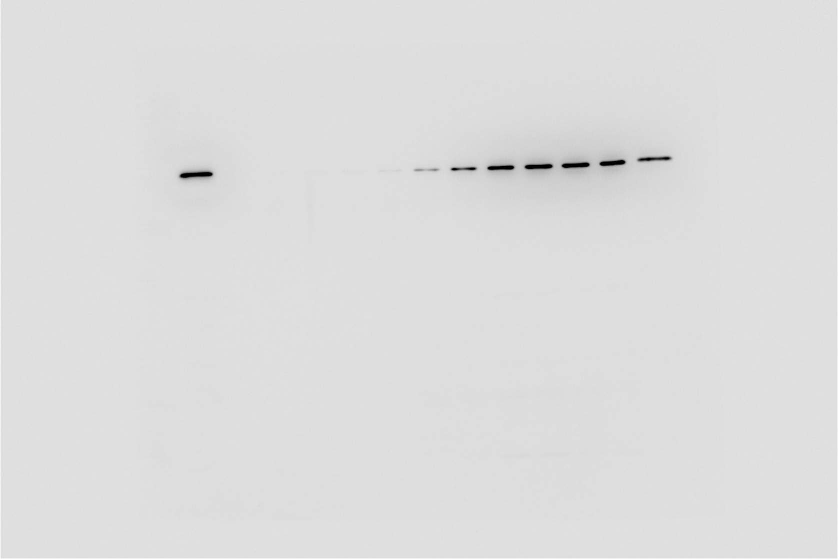

WB : αeIF4G

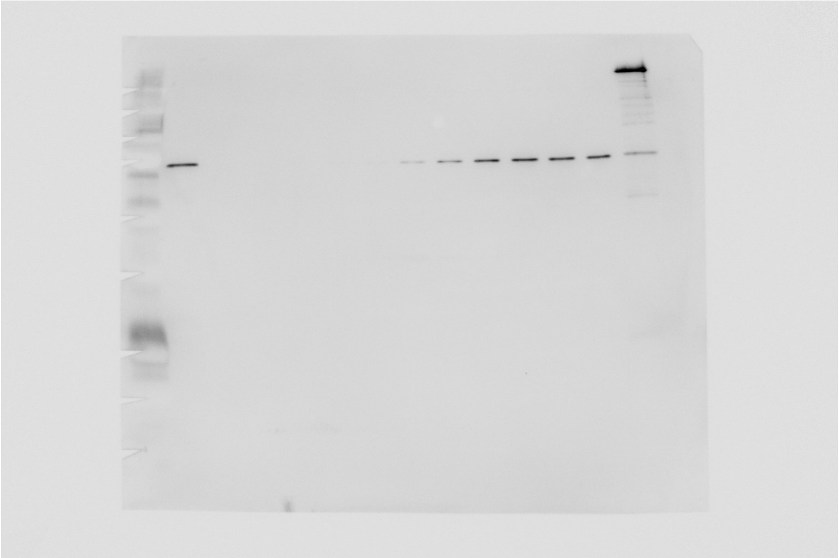

Unprocessed images

Figure 1, F

WB : αPABP

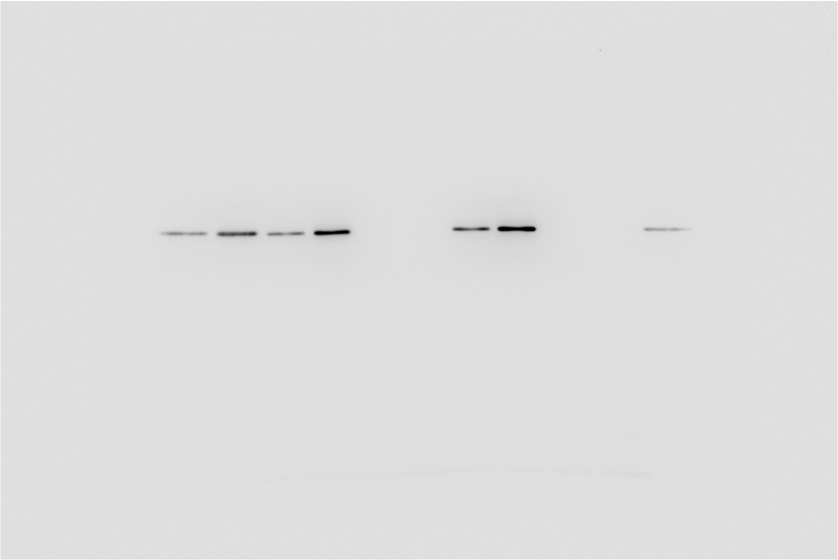

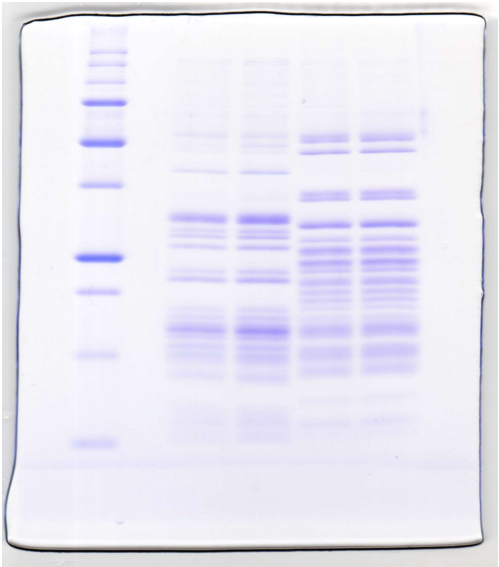

Unprocessed images

Figure 2, A

PABP

WB : αPABP

40S+PABP

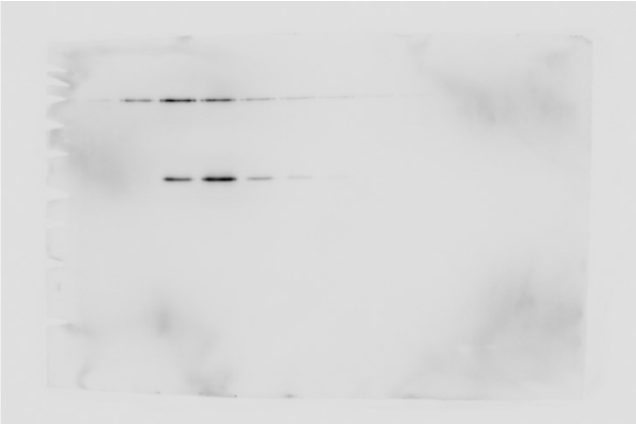

WB : αPABP

WB : αS6

60S+PABP

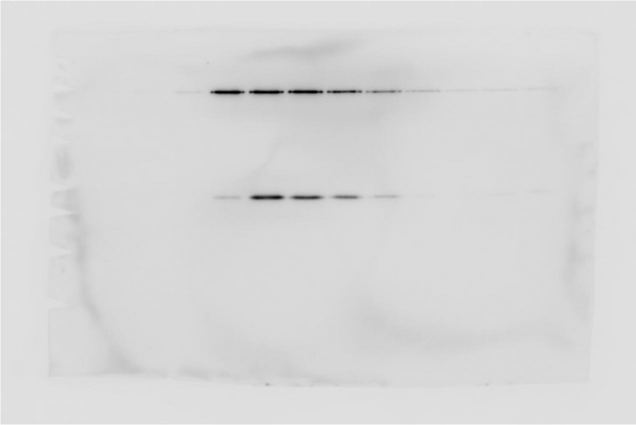

WB : αPABP

WB : αL13a

Unprocessed images

Figure 2, B

40S+PABP+poly(A)

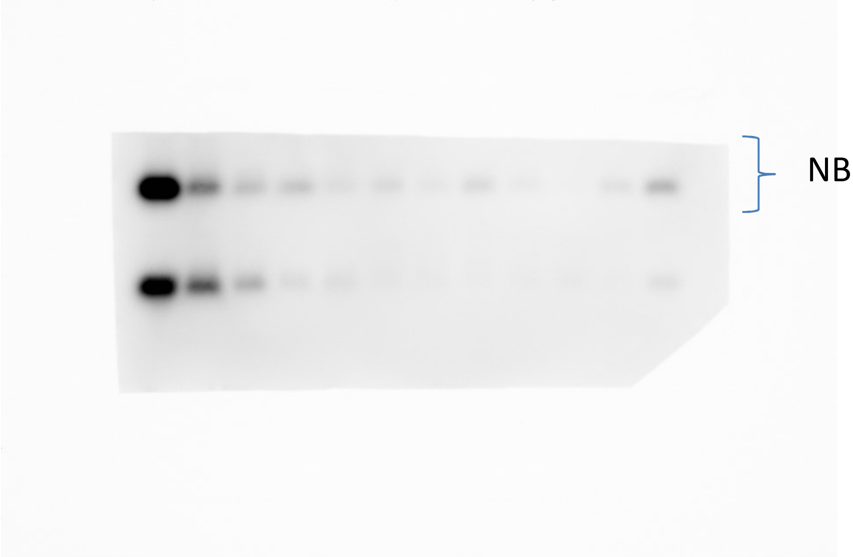

NB : αpoly(A)

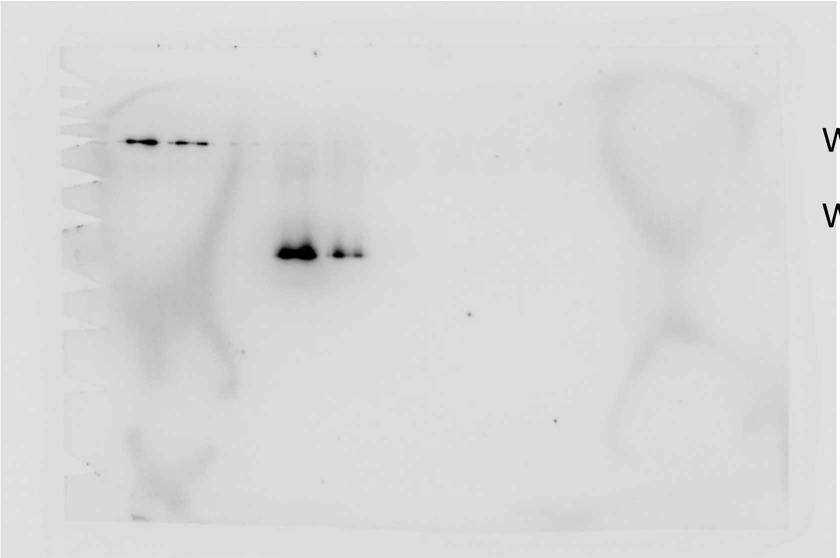

WB : αPABP

WB : αS6

Unprocessed images

Figure 2, B

60S+PABP+poly(A)

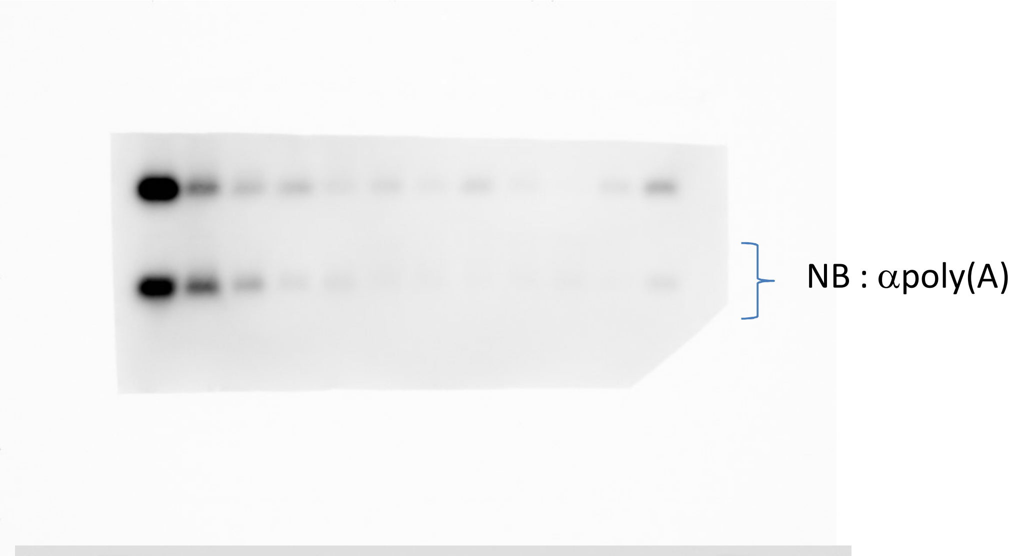

NB : αpoly(A)

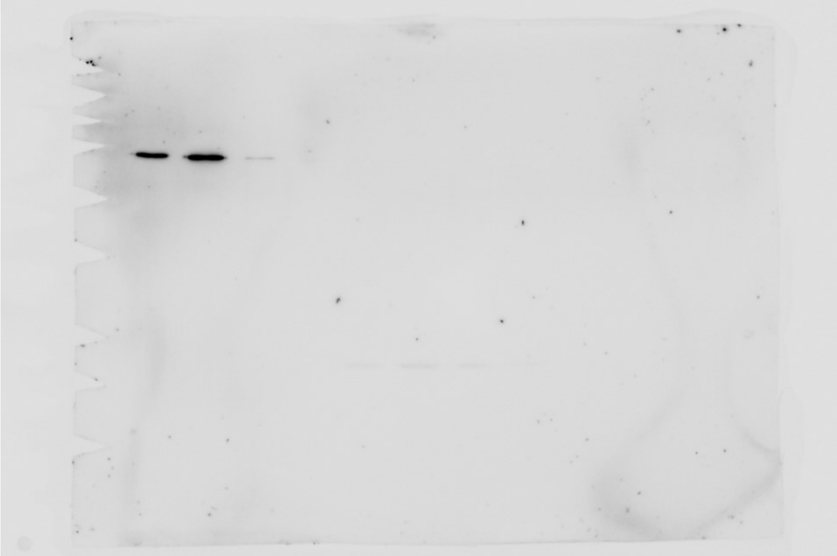

WB : αPABP

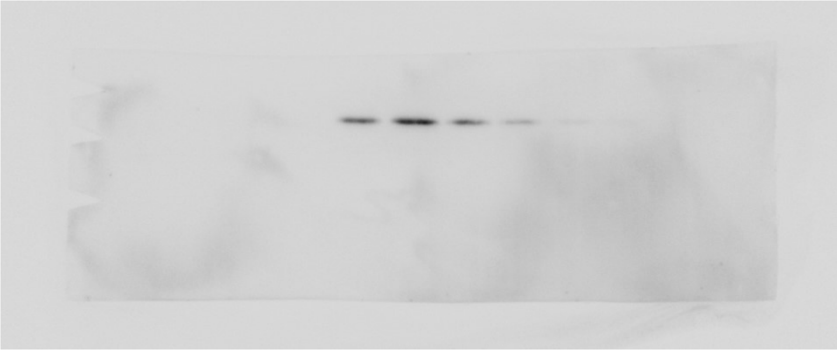

WB : αL13a

Unprocessed images

Figure 2, C

40S+PABP+HA-Rluc-N

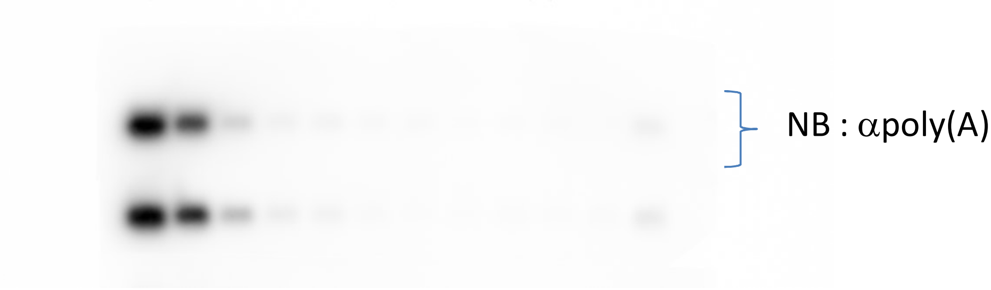

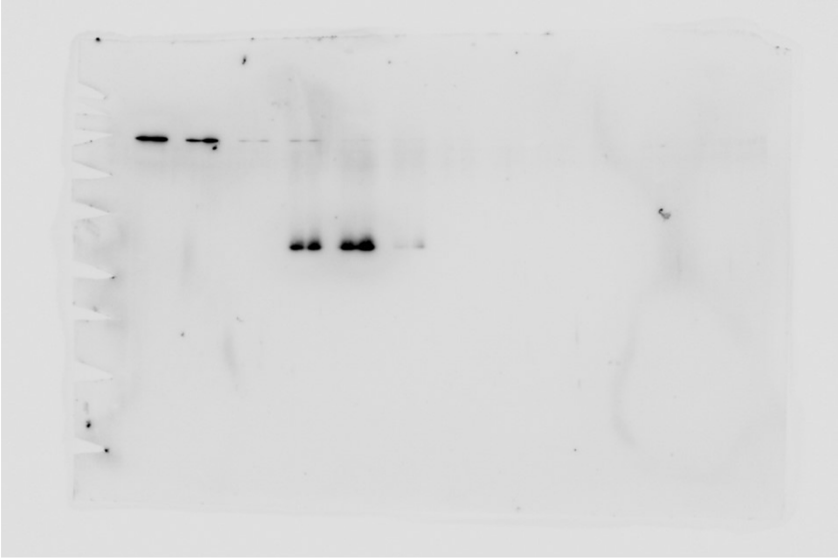

Unprocessed images

Figure 2, C

60S+PABP+HA-Rluc-N

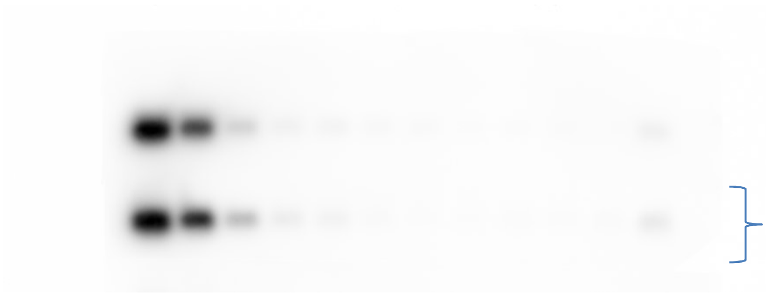

NB : αpoly(A)

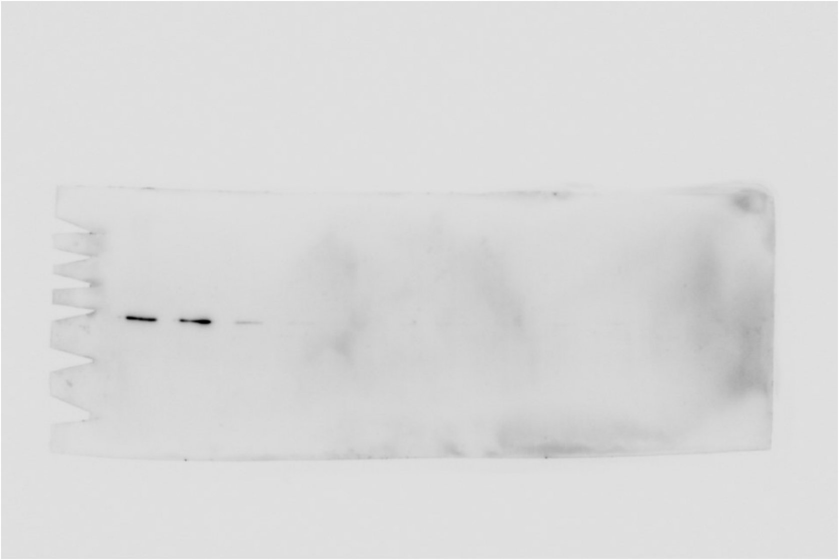

WB : αPABP

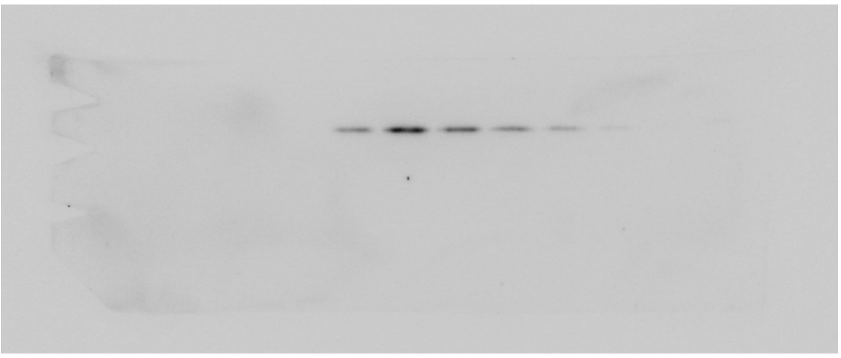

WB : αL13a

Unprocessed images

Figure 2, D

PABP+40S, poly(A)

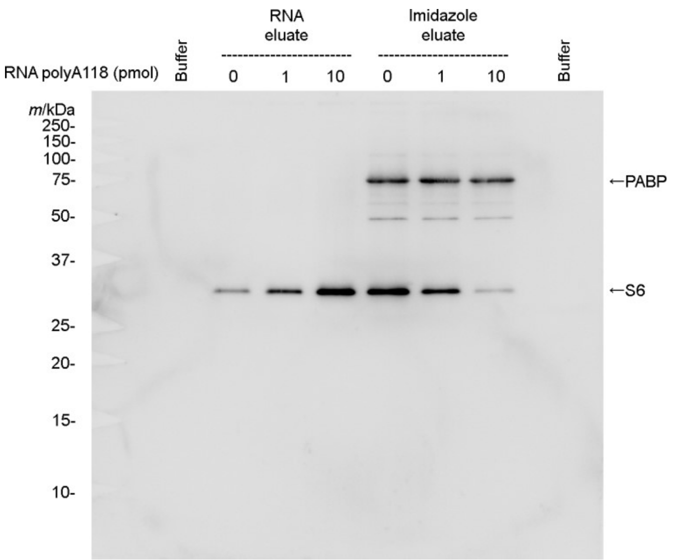

PABP+40S, HA-Rluc-N

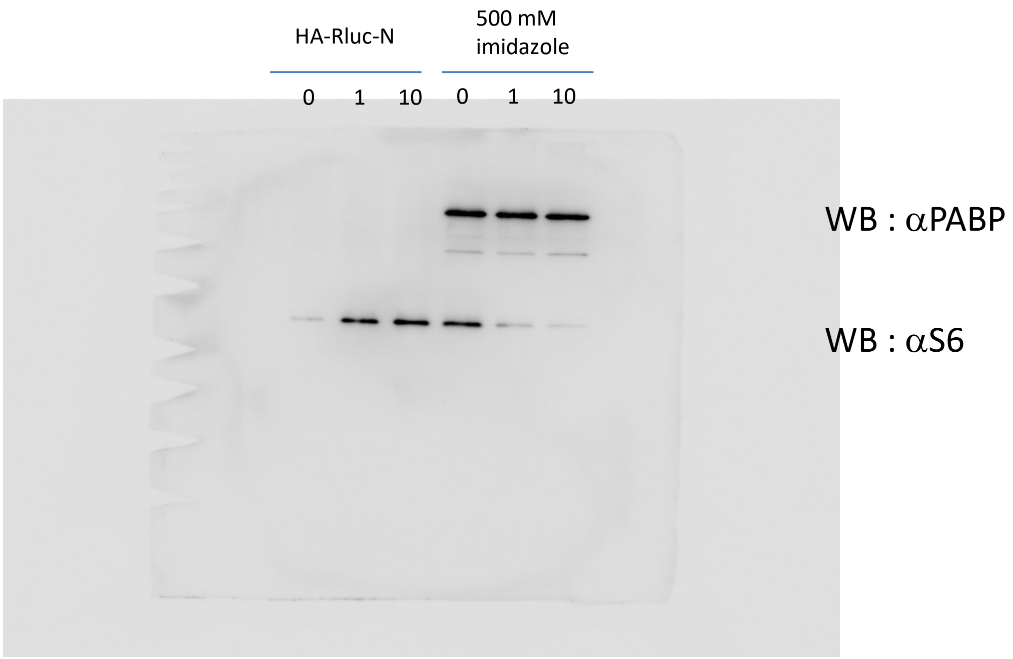

Unprocessed images

Figure 2, D

PABP+60S, poly(A)

Poly(A) — 500 mM imidazole

0 1 10 0 1 10

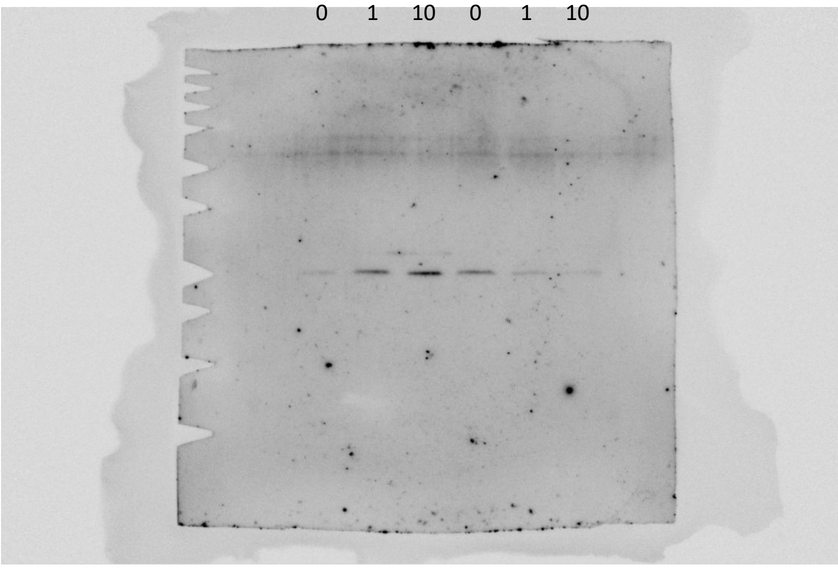

WB : αL13a

Poly(A) — 500 mM imidazole

0 1 10 0 1 10

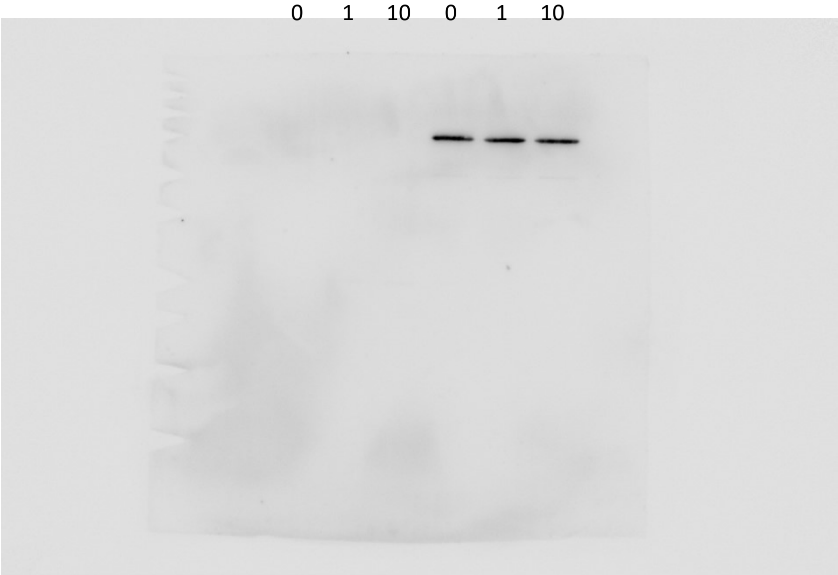

WB : αPABP

Unprocessed images

Figure 2, D

PABP+60S, HA-Rluc-N

HA-Rluc-N 500 mM imidazole

0 1 10 0 1 10

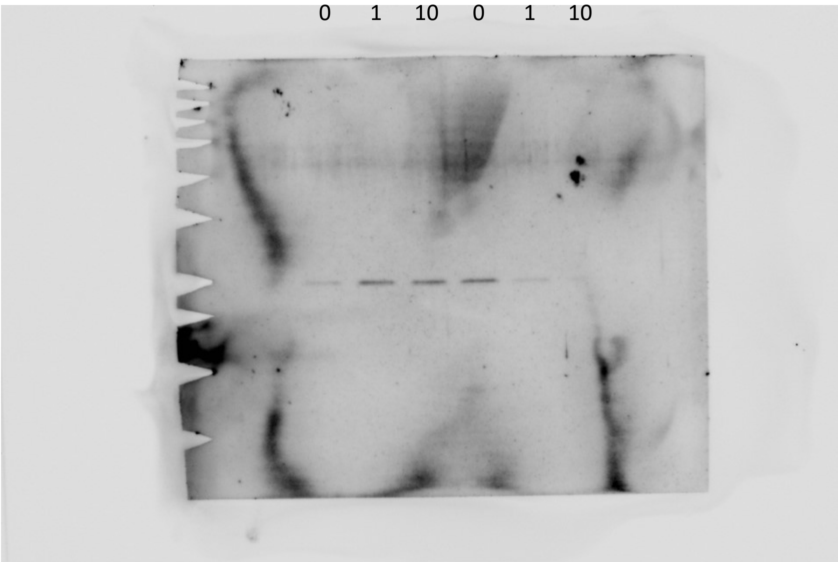

WB : αL13a

HA-Rluc-N 500 mM imidazole

0 1 10 0 1 10

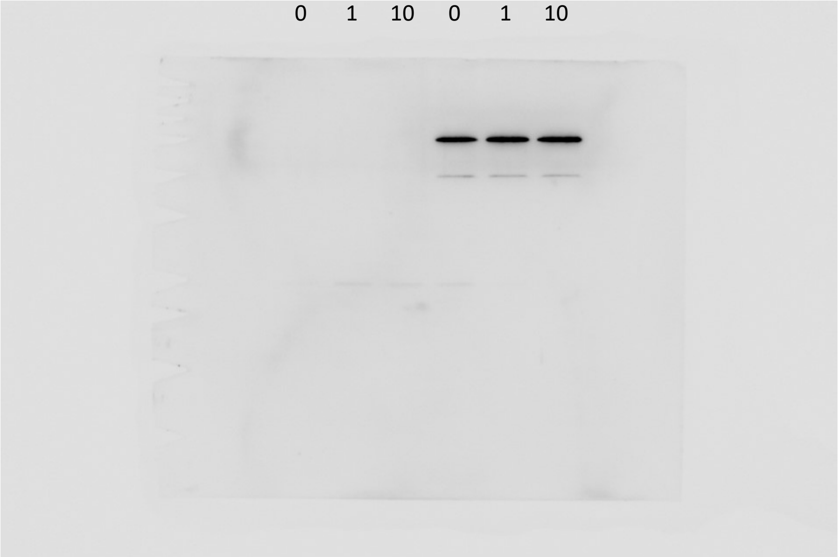

WB : αPABP

Unprocessed images

Figure 3, B

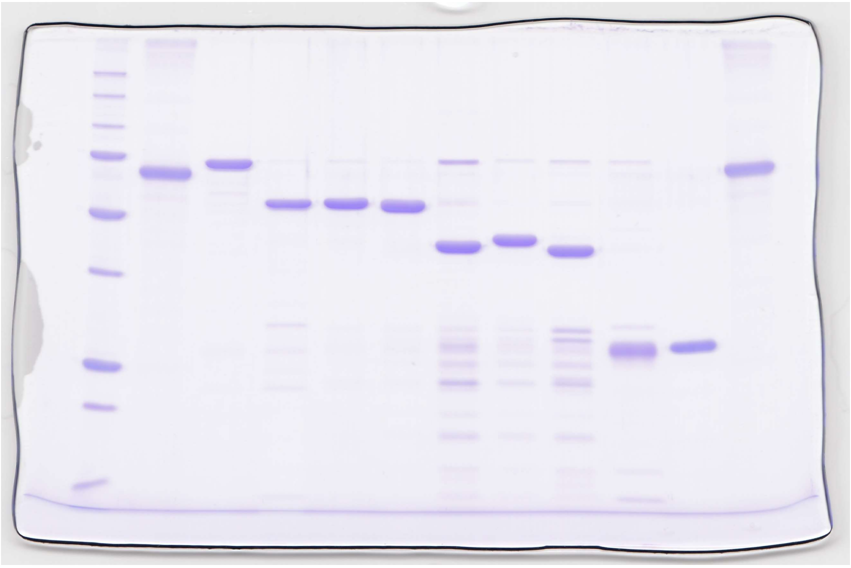

Unprocessed images

Figure 3, C

WB : αS6

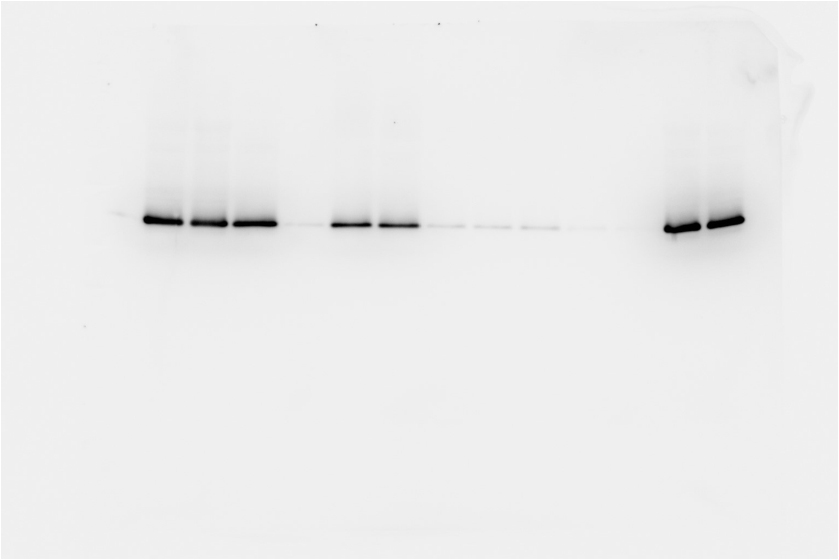

WB : αPA

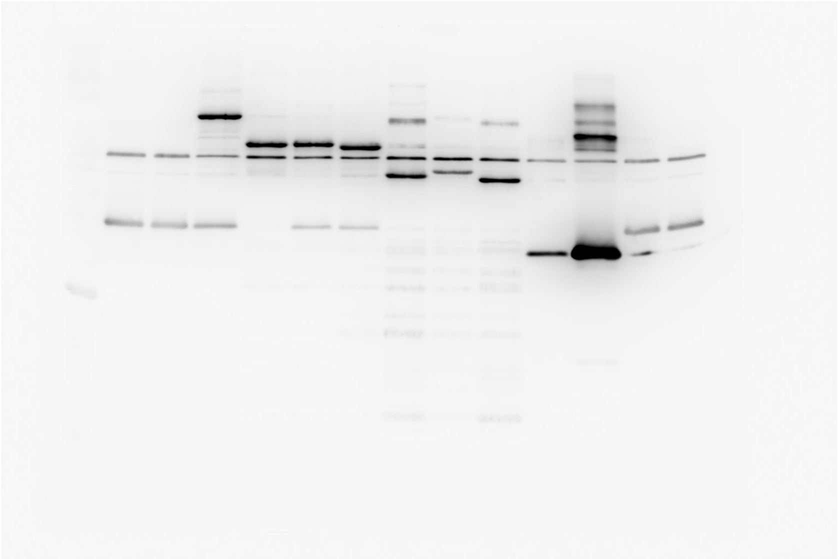

Unprocessed images

Figure 3, D

WB : αL13a

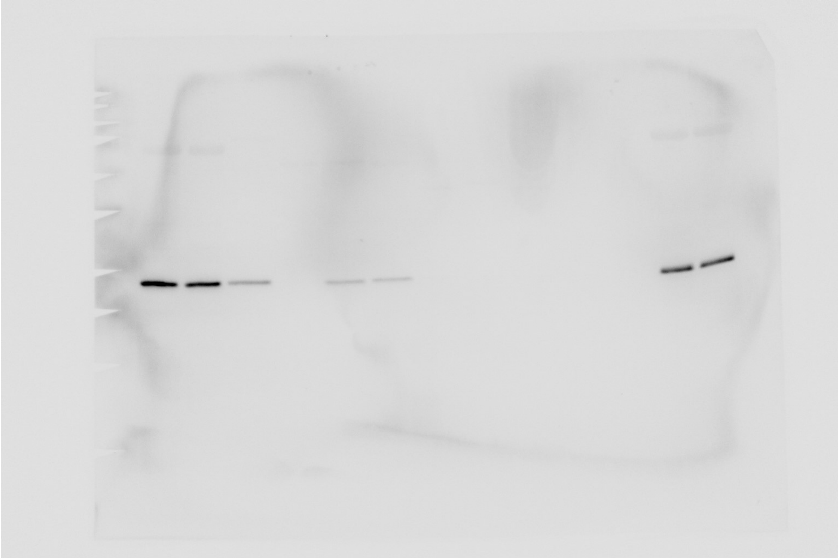

WB : αPA

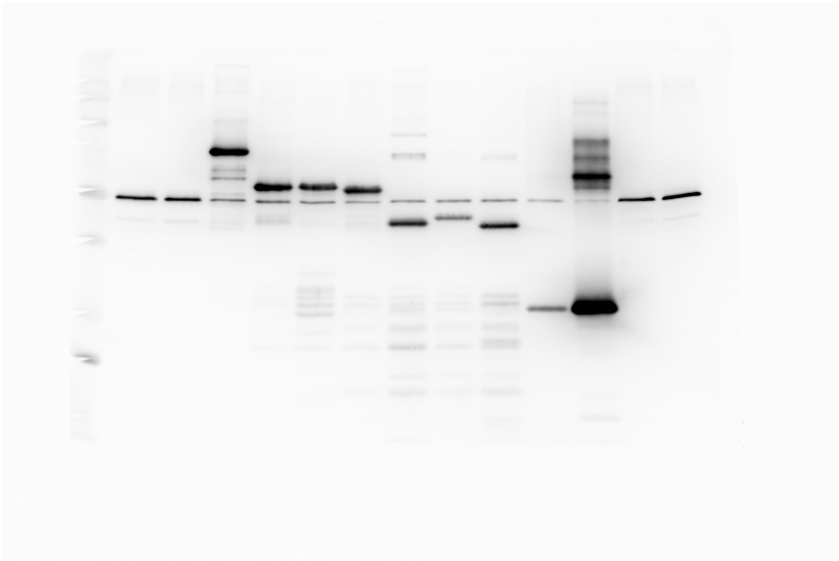

Unprocessed images

Figure 5, A

WB : αMyc

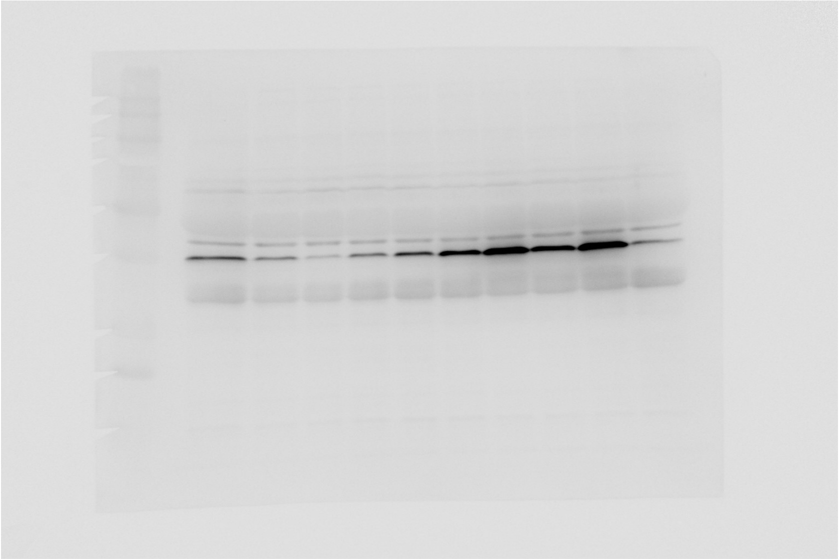

Unprocessed images

Figure S1, A

Upper
WB : αS6

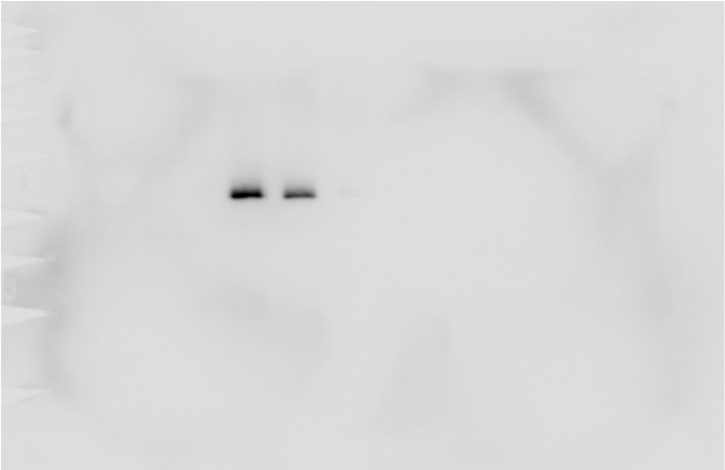

Lower
WB : αL13a

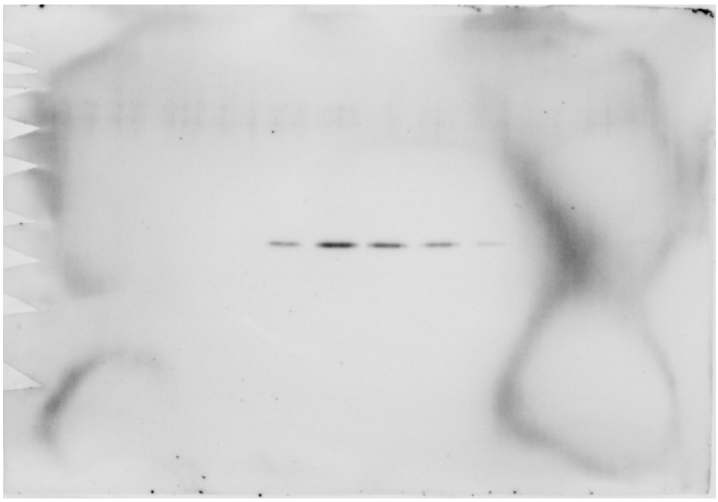

Unprocessed images

Figure S1, B

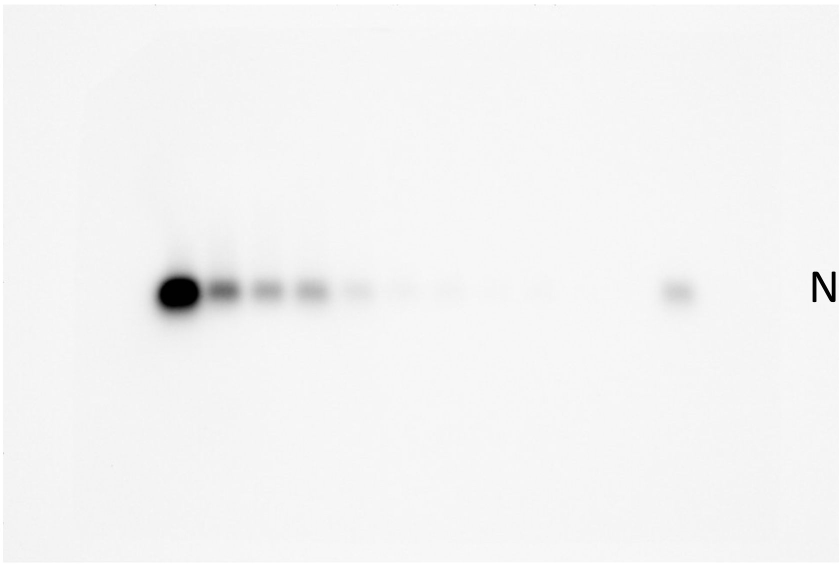

NB : αpoly(A)

Unprocessed images

Figure S1, B

PABP+poly(A)

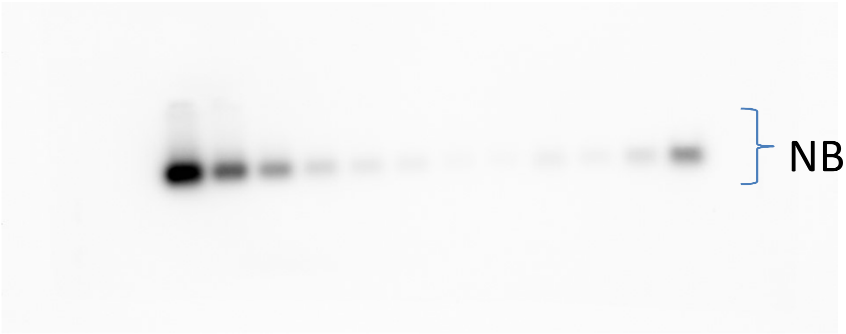

NB : αpoly(A)

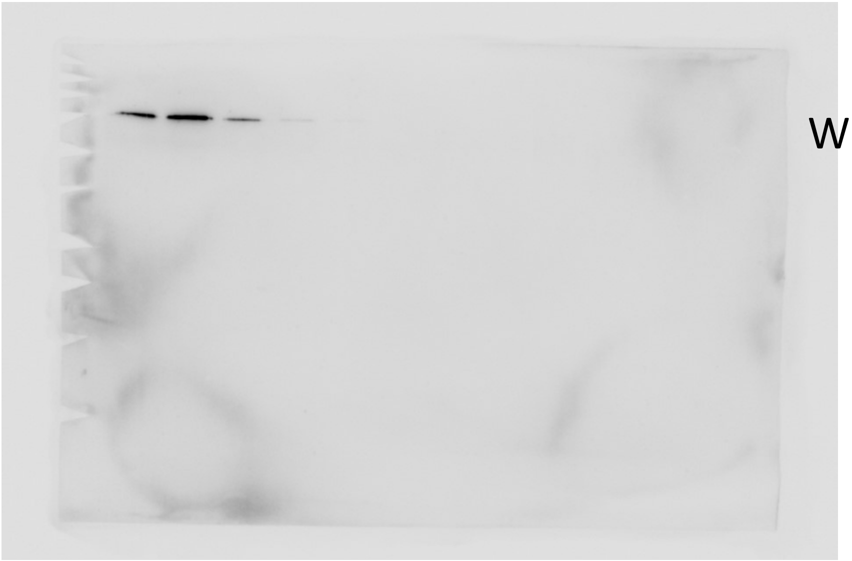

WB : αPABP

Unprocessed images

Figure S1, B

40S+poly(A)

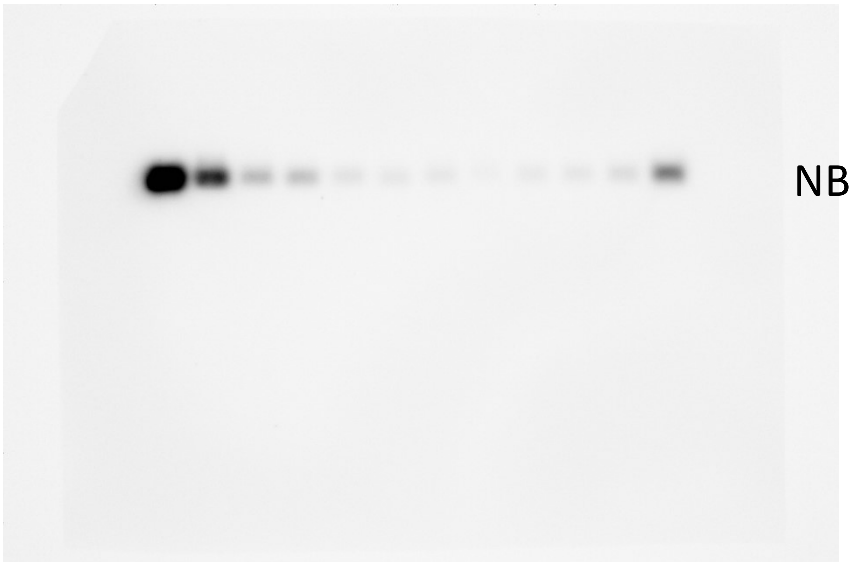

NB : αpoly(A)

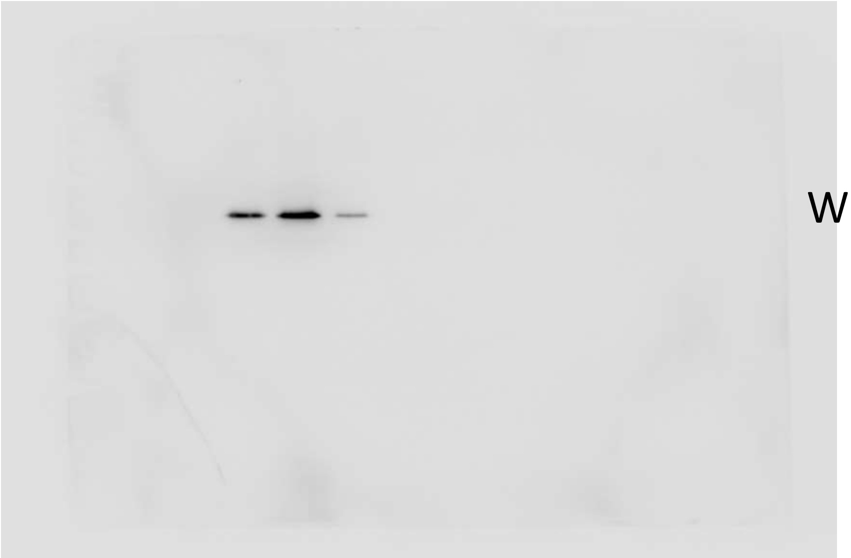

WB : αS6

Unprocessed images

Figure S1, B

60S+poly(A)

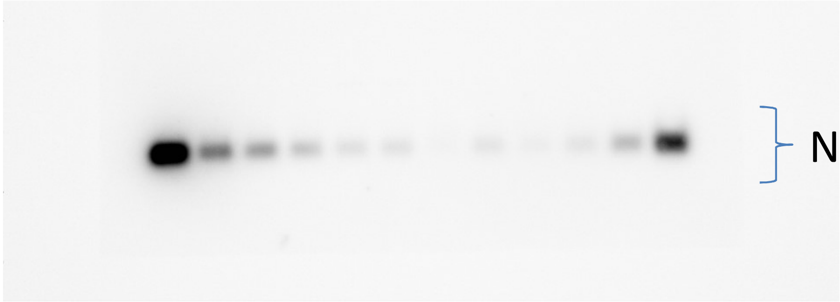

NB : αpoly(A)

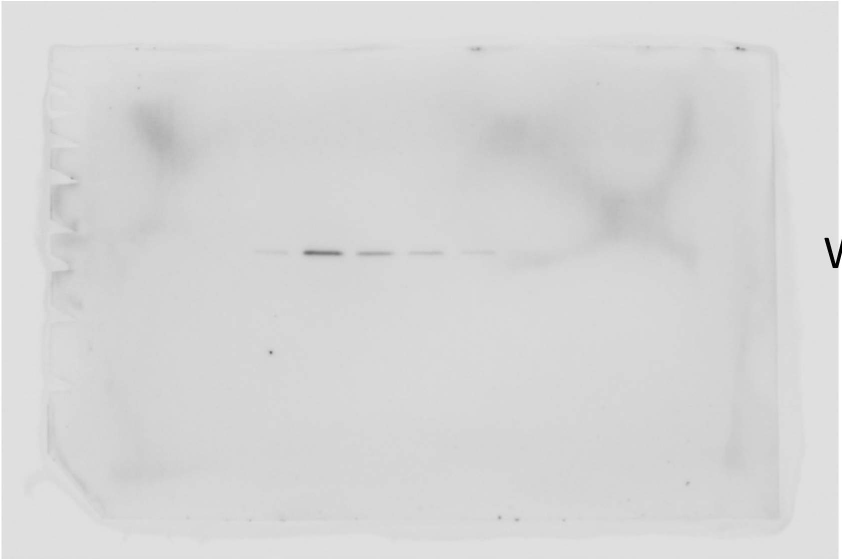

WB : αS6

Unprocessed images

Figure S1, C

40S+eIF4G/4E+PABP

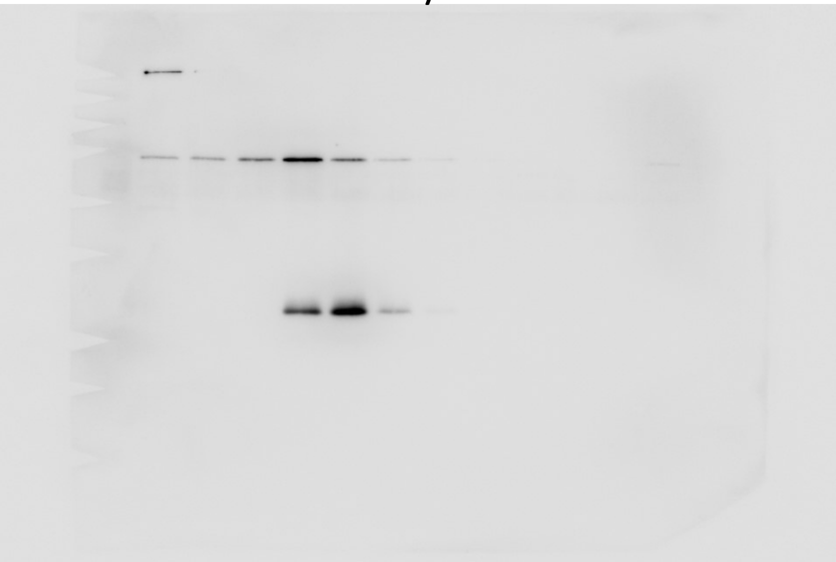

WB : αFLAG(eIF4G)

WB : αPABP

WB : αS6

60S+eIF4G/4E+PABP

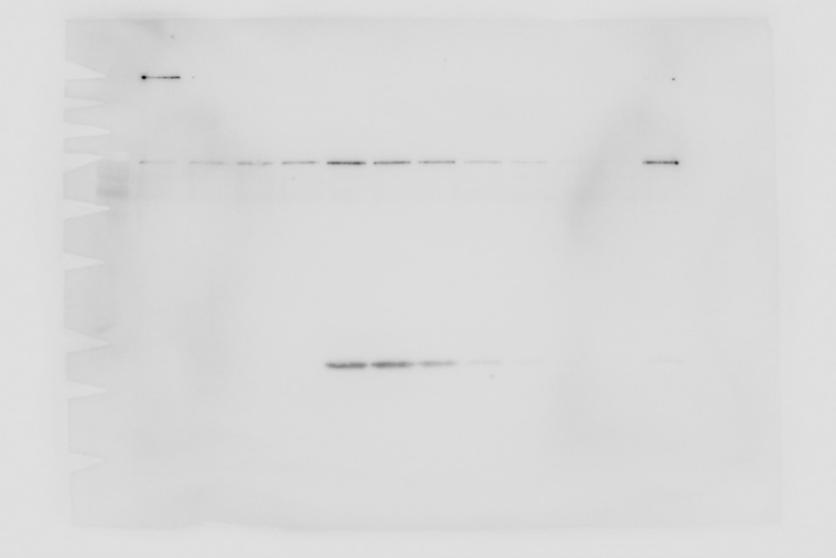

WB : αFLAG(eIF4G)

WB : αPABP

WB : αL13a

Unprocessed images

Figure S3

eIF1, 1A, 2B, 4A, 4B, 4E/4G, 5, 5B, DHX29, PABP (13% gel)

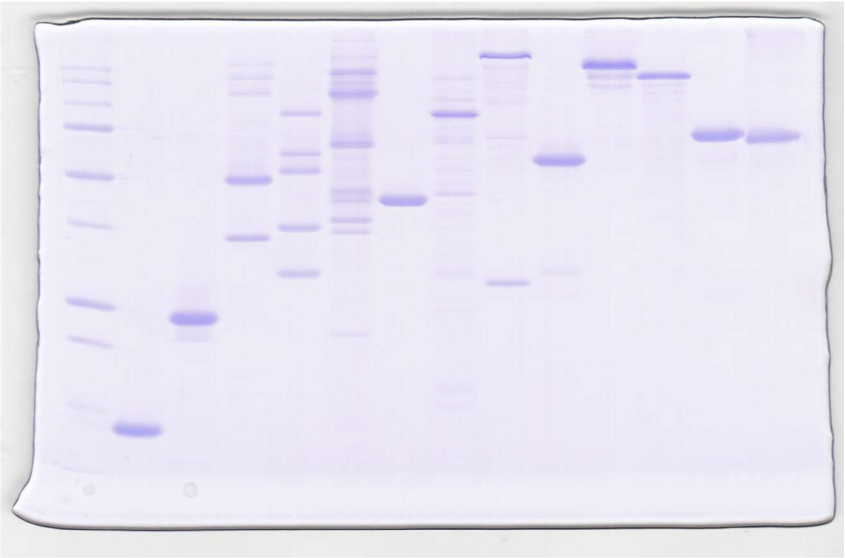

eIF2, 3 (4-12% NuPAGE gel)

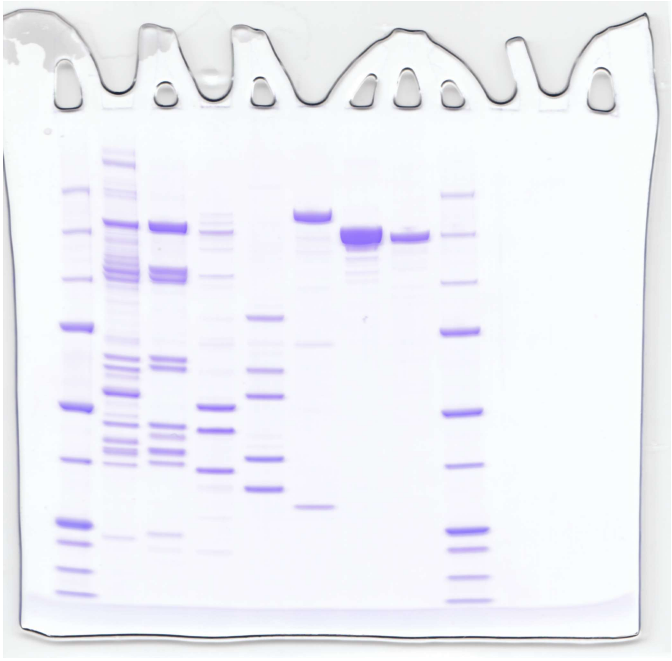

Supplement: Supplementary file 1 — Supplementary information [file 41598_2018_35753_MOESM1_ESM.pdf]
